# AraC‐Family Transcriptional Regulator WhpR Controls Virulence in *Pseudomonas savastanoi* pv. savastanoi Through Regulation of Indole Metabolism

**DOI:** 10.1111/1751-7915.70247

**Published:** 2025-10-21

**Authors:** Antonio Arroyo‐Mateo, Jesús Leal‐López, Luis Rodríguez‐Moreno, Cayo Ramos

**Affiliations:** ^1^ Área de Genética, Facultad de Ciencias Universidad de Málaga Málaga Spain; ^2^ Instituto de Hortofruticultura Subtropical y Mediterránea “La Mayora” Consejo Superior de Investigaciones Científicas (IHSM‐UMA‐CSIC) Málaga Spain

**Keywords:** indol‐3‐acetic acid, indole metabolism, olive plants, *Pseudomonas savastanoi*, *Pseudomonas syringae*, tryptophan, virulence, WHOP region, woody host

## Abstract

The *Pseudomonas syringae* complex WHOP genomic island underpins virulence in woody hosts by mediating the catabolism of aromatic compounds. However, the biochemical functions of the *ipoABC* and *dhoAB* operons and the regulatory gene *whpR* remain unknown. Comparative genomics revealed WHOP‐like clusters beyond *P. syringae* , found in diverse plant‐associated, environmental and clinical bacteria, including indole degraders. We propose that *ipoABC* and *dhoAB* mediate indole degradation via anthranilate, linking indole detoxification to central metabolism through the β‐ketoadipate pathway. In the olive pathogen *P. savastanoi* pv. savastanoi, *ipoABC* promotes indole degradation, indigo production, cell aggregation and biofilm formation. WhpR, an AraC‐family regulator structurally related to CuxR and ToxT, defines a regulon comprising repression of most WHOP operons along with genes outside this region, including *trpAB*, reflecting integrated regulation of indole catabolism and tryptophan biosynthesis. In line with the observed transcriptional repression of WHOP genes, deletion of *whpR* led to hypervirulence and significantly altered bacterial fitness in woody olive plants. These findings define the WHOP region as a regulatory hub linking indole detoxification, multicellular behaviour and virulence, emerging as a target for novel control strategies against woody plant diseases.

## Graphical abstract

The WHOP genomic island of *Pseudomonas syringae* complex pathogens integrates indole degradation, multicellular behaviour, fitness and virulence regulation in woody hosts. We propose that the *ipoABC* and *dhoAB* operons mediate indole catabolism to anthranilate, which is channeled into the tricarboxylic acid cycle (TCA). In the olive knot pathogen *Pseudomonas savastanoi* pv. savastanoi, the AraC‐family regulator WhpR represses WHOP genes and links indole degradation to the biosynthesis of tryptophan, the precursor of the virulence‐associated phytohormone indole‐3‐acetic acid (IAA).

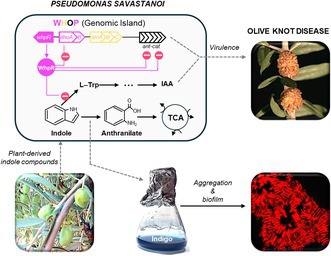

## 1 Introduction

*Pseudomonas savastanoi* , a member of the *Pseudomonas syringae* complex, induces excrescences or knots in woody plants and infects some herbaceous species. It belongs to phylogroup 3 (PG3), which includes over 25 distinct pathovars and four additional *Pseudomonas* species. *P. savastanoi* encompasses several pathovars whose strains have been isolated from various woody hosts, such as pv. fraxini (Psf) from ash ( *Fraxinus excelsior* ), pv. retacarpa (Psr) from broom (*Retama sphaerocarpa*), pv. mandevillae (Psm) from dipladenia (*Mandevilla* spp.), pv. nerii (Psn) from oleander ( *Nerium oleander* ) and pv. savastanoi (Psv) from olive ( *Olea europaea* ) (Gardan et al. 1992; Bull et al. 2010; Moreno‐Pérez et al. 2020; Caballo‐Ponce et al. 2021). Importantly, tree‐infecting *P. savastanoi* pathovars are more closely related to other PG3 *P. syringae* pathovars associated with trees than to herbaceous‐infecting *P. savastanoi* pathovars, such as pv. phaseolicola and pv. glycinea, which cause bean halo blight and soybean brown spot, respectively (Gardan et al. 1999; Nowell et al. 2014; Baltrus et al. 2017). PG3 is the only phylogroup in the *P. syringae* complex that harbors additional tumour‐inducing bacteria infecting woody hosts. These include several *P. syringae* pathovars whose strains have been isolated from cherry ( *Prunus yedoensis* ), bayberry ( *Myrica rubra* ) and kakuremino (*Dendropanax trifidus*), among others, as well as *Pseudomonas meliae* (chinaberry, *Melia azedarach* ) and *Pseudomonas tremae* (trema, *Trema orientalis* ; Lamichhane et al. 2014).

Research into the molecular basis of tree diseases has intensified in recent years, highlighting the role of specific virulence factors that enable pathogens to colonise and infect woody hosts. Pathogenicity and virulence of *P. savastanoi* pathovars of woody hosts rely on a type III secretion system (T3SS) and an effectorome comprising at least 45 T3SS effectors. Of note, effectors such as HopAY1, HopAO1, HopBL1 and HopBL2 are strongly associated with virulence in woody plants (Matas et al. 2014; Nowell et al. 2016; Caballo‐Ponce, Murillo, et al. 2017; Caballo‐Ponce, Van Dillewijn, et al. 2017; Moreno‐Pérez et al. 2020). Successful symptom development also requires the bacterium to maintain optimal levels of various metabolites, including phytohormones such as cytokinins (Añorga et al. 2020) and indole‐3‐acetic acid (IAA) (Aragón et al. 2014; Pintado et al. 2023), as well as cyclic‐di‐GMP (Aragón, Pérez‐Mendoza, Gallegos, and Ramos 2015; Aragón, Pérez‐Mendoza, Moscoso, et al. 2015). These metabolites regulate the expression of additional virulence‐related genes and enhance bacterial competitiveness. Quorum sensing molecules further facilitate communication between *P. savastanoi* and other members of the knot microbiome, playing a critical role in tumour formation (Caballo‐Ponce et al. 2018). In addition, Ca^2+^ entry and signalling (Moretti et al. 2019) as well as the global regulatory system GacS/GacA (Lavado‐Benito et al. 2024) integrate environmental cues with bacterial virulence mechanisms, further contributing to *P. savastanoi* pathogenicity.

A distinctive feature of *P. savastanoi* and other *P. syringae* complex bacteria isolated from woody tissues is the presence of a 15 kb genomic island, termed WHOP (from woody host and *Pseudomonas*; Caballo‐Ponce, Van Dillewijn, et al. 2017). This island is found exclusively in PG1 and PG3 strains from woody hosts and is encoded in strains of all PG3 tumorigenic species and pathovars except for *P. tremae* . The WHOP region contains four operons and three independently transcribed genes, most of which contribute to the breakdown of phenolic compounds (Caballo‐Ponce, Van Dillewijn, et al. 2017). In woody plants, these substrates include not only lignin‐derived aromatics but also indole‐derived defense metabolites, which can exhibit antibacterial activity (Stahl et al. 2016; Li et al. 2023). Such compounds may constitute key targets of WHOP‐encoded functions, raising the question of whether indolic compounds available *in planta* are predominantly plant‐derived or also produced by *P. syringae* during infection of woody hosts.

In Psv strain NCPPB 3335, the *antABC* and *catBCA* operons mediate the catabolism of anthranilate and catechol, respectively, and contribute to virulence on olive plants. The *ipoABC* operon, also required for full Psv virulence, is linked to oxygenase activity on aromatic compounds. Although the function of the *dhoAB* operon remains unclear, mutants lacking this operon display reduced competitive growth in planta. Among the independent genes, *antR* regulates the *antABC* operon, while PSA3335_RS13065 encodes a putative aerotaxis receptor essential for the full fitness of Psv in olive plants. The role of the third independently transcribed gene (PSA3335_RS13035) is still undetermined. This gene, located near the *dhoAB* operon but oriented oppositely, is annotated as *benR*, a regulator of the *benABCD* operon found in other *Pseudomonas* spp., which is absent in *P. savastanoi* strains (Caballo‐Ponce, Van Dillewijn, et al. 2017).

In this study, we analysed the distribution of genes homologous to those encoded in the *P. syringae* WHOP region (WHOP‐like clusters) across bacterial genomes and examined their predicted functions, focusing on pathways involved in indole degradation. We also investigated the structure and regulatory potential of the *P. savastanoi* BenR homologue, designated WhpR (WHOP regulator) and explored its role in coordinating expression of the WHOP region and a subset of genes encoded outside the cluster. These analyses provide a framework to understand how WHOP‐encoded pathways, together with both bacterial and plant‐derived indole compounds, may influence the virulence and ecological adaptation of bacterial phytopathogens to woody hosts.

## 2 Experimental Procedures

### 2.1 Strain, Plasmids and Growth Conditions

Bacterial strains, plasmids and primers used in this work are listed in Tables S1, S2 and S3. *P. savastanoi* strains were cultured at 28°C in lysogeny broth (LB) (Lennox 1955), M9 medium containing 5 mM succinate and 5 g/L NH_4_Cl or Hrp‐Inducing Medium (HIM) (Huynh et al. 1989). *Escherichia coli* strains were grown in LB at 37°C. Antibiotics were added, as required, at the following concentrations (mg/mL): for *P. savastanoi* : ampicillin (Ap) 400, gentamicin (Gm) 10, kanamycin (Km) 7, nitrofurantoin (Nf) 25 and cycloheximide (Ch) 100. For *E. coli* : Ap 100, Gm 10 and Km 50.

### 2.2 Bioinformatics, Structural Predictions and Functional Annotation

**FIGURE 1:**
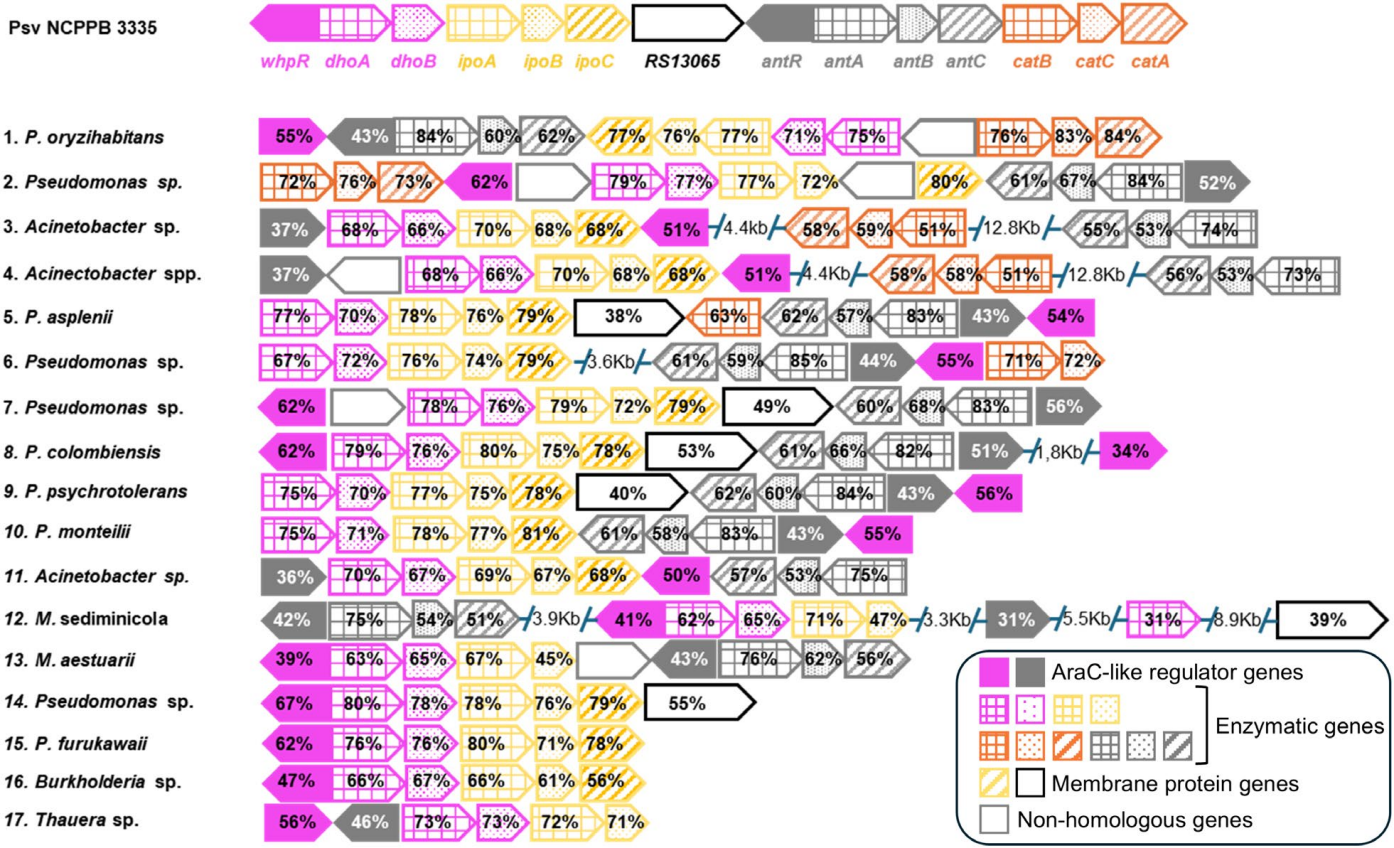
WHOP‐like clusters in bacterial genomes identified by MultiGeneBlast. The WHOP region of *P. savastanoi* pv. savastanoi (Psv) NCPPB 3335 was used as the query (top line). Genes are shown as arrows, colour‐coded by operon membership. Homologous genes share the same colours and patterns as their counterparts in the query. Percentages within the arrows denote amino acid identity with the corresponding Psv NCPPB 3335 orthologue. Clusters are ranked (1–17) according to their Total and Cumulative MultiGeneBlast scores against the query. Bacterial strains within each cluster are indicated by species abbreviation (see Table S4). Gene categories are indicated in the visual key. Membrane protein genes include *ipoC* and the putative aerotaxis receptor gene *RS13065*.

Enzymatic functions encoded in the *P. syringae* WHOP region were predicted using the Kyoto Encyclopedia of Genes and Genomes (KEGG) via KO term assignment through blastKOALA (Kanehisa, Sato, Kawashima, et al. 2016; Kanehisa, Sato, and Morishima 2016). Genes homologous to the WHOP region of Psv NCPPB 3335 (WHOP‐like clusters) were identified using MegaBLAST (Zhang et al. 2000) on the National Center for Biotechnology Information (NCBI) server with an *e*‐value threshold of < 5 × 10^−5^. Additionally, MultiGeneBlast (Medema et al. 2013) was used for the identification, considering both nucleotide sequence similarity and gene synteny. To build the search database, the *whpR* gene from Psv NCPPB 3335 (*e*‐value < 5 × 10^−5^) was initially queried in MegaBLAST. After excluding *P. syringae* complex strains, hits from other *Pseudomonas* species and representatives of the genera *Acinetobacter, Burkholderia*, *Cupriavidus* and *Marinobacterium* were compiled into a database for MultiGeneBlast analysis. A homology search using the entire WHOP region as a query was performed under default settings. Positive hits were manually curated for the presence of *whpR*, the complete *dhoAB* operon, and at least the *ipoA* and *ipoB* genes. Seventy‐eight selected genomes were examined for WHOP gene organisation, *ipoC* presence and gene orientation, revealing 17 different WHOP‐like clusters. Genomes were ranked based on Total and Cumulative scores, which accounted for orthologous protein number, gene synteny, sequence similarity and alignment quality. Representative genomes with the highest scores were selected for each cluster (Figure 1, Table S4).

Protein family classification, domain prediction and functional site annotation were conducted with InterPro (Blum et al. 2025). Structural homologues of WhpR were identified using I‐TASSER (Zhou et al. 2022), a hierarchical approach to protein structure prediction and structure‐based function annotation. To model the 3D structures of the proteins, AlphaFold 3 (Abramson et al. 2024) was employed. *In silico* ligand‐docking analyses of WhpR with indole and anthranilate were performed using SwissDock 2 (Bugnon et al. 2024), employing the AutoDock Vina method (Eberhardt et al. 2021). The generated images were visualised and edited with ChimeraX (Pettersen et al. 2021).

### 2.3 Construction of *P. savastanoi* Mutants and Complemented Strains

The *whpR* gene was deleted from the Psv NCPPB 3335 genome using plasmid p*whpR*‐Km, constructed in the pGEM‐T backbone by cloning fragments of approximately 1 kb corresponding to the genomic sequences flanking *whpR* on both sides, with the *nptII* gene conferring kanamycin resistance inserted between them (Tables S1 and S2). Plasmid transformation and selection of the mutants followed the protocols described by Pérez‐Martínez et al. (2007) and Matas et al. (2014), respectively. The kanamycin resistance marker was subsequently excised using plasmid pFLP2 (Table S2).

For complementation, the *whpR* coding region was PCR‐amplified from Psv NCPPB 3335, verified for accuracy and cloned into the pAMEX (Km^R^) vector under the control of the *nptII* promoter, generating pAMEX:*whpR* (Table S2).

### 2.4 Indole Utilisation Assays

Indole degradation was assessed using a 5‐bromoindoline plate assay, in which this indole analogue produces a purple compound upon degradation (Sadauskas et al. 2017). Psv strains grown on LB plates were streaked onto LB plates supplemented with 1 mM 5‐bromoindoline and incubated at 28°C for 48 h until pigment development was visible.

For indole utilisation assays in M9 minimal medium, Psv cells grown overnight on M9 were transferred to 100 mL flasks containing 1 mM indole, adjusting the initial OD600 to 0.05. After 24 h of incubation, samples were collected for confocal microscopy and flow cytometry analyses. Experiments were performed independently three times, each with three biological replicates.

### 2.5 Confocal Microscopy

A Zeiss LSM 880 confocal microscope was used. For each strain, 100 μL aliquots from 24‐h M9 cultures grown with or without 1 mM indole were prepared. Cells were stained with FM4‐64 membrane dye following manufacturer recommendations and incubated for 5 min at room temperature. Images were acquired and processed using ZEN Blue 3.11 software.

### 2.6 Flow Cytometry

Flow cytometry of cell size and complexity was performed on M9 cultures, with and without 1 mM indole, incubated for 24 h. Up to 10,000 events of undiluted culture were recorded using a FACSverse cytometer. Experiments were independently repeated three times, with data analysed using Kaluza 2.3 software.

### 2.7 RNA Extraction

*P. savastanoi* strains were cultured overnight in LB medium at 28°C and diluted to an OD600 of 0.1 in fresh LB medium. Cultures were distributed into three biological replicates (110 mL each) and incubated until mid‐exponential phase (OD600 ≈0.5). Cells from each replicate were harvested by centrifugation, washed and resuspended in 48 mL of HIM medium (Huynh et al. 1989). After 6 h of incubation at 28°C, cells were collected and aliquoted into six 1 mL samples of the same medium. Cell pellets were obtained by centrifugation at 4°C, snap‐frozen in liquid nitrogen and stored at −80°C until RNA extraction.

Total RNA was extracted using the Direct‐zol RNA MiniPrep kit (Zymo Research, CA, USA) according to the manufacturer's instructions. Residual genomic DNA was removed using the TURBO DNA‐free kit (Invitrogen, CA, USA).

### 2.8 RNA‐Seq Sequencing and Data Processing

Sequencing libraries were prepared by first depleting ribosomal RNA (rRNA) using the Ribo‐Zero Plus kit (Illumina) to minimise rRNA contamination. RNA was quantified using the Qubit 3.0 Fluorometer (Life Technologies), and its integrity was assessed by agarose gel electrophoresis. Quality control was performed using the Bioanalyzer 2100 (Agilent) with the 2100 Expert software (version B.02.08.SI648), using the Prokaryote RNA Pico assay. RNA purity was evaluated on a NanoVue Plus (GE Healthcare) via A_260/280_ and A_260/230_ ratios. Total RNA from three samples of the wild‐type strain Psv NCPPB 3335 and three from its Δ*whpR* mutant was sequenced on the NextSeq 550 platform (Illumina) at the Supercomputing and Bioinnovation Centre (SCBI) of the University of Málaga (UMA) using a 150‐cycle paired‐end run (2 × 74 bp) configuration.

The raw RNA‐seq reads were uploaded to the Sequence Read Archive (SRA) database under the reference SUB14630104 and processed at the SCBI. Quality control and sequence cleaning were performed using fastp (Chen et al. 2018), applying a minimum length threshold of 40 nucleotides to remove adapters and short reads. The processed sequences were mapped to the reference genome of Psv NCPPB 3335 using Bowtie 2 (Langmead and Salzberg 2012), incorporating the complete sequences of its chromosome (GCF_000164015) and its three native plasmids: pPsv48A (NC_019265.2), pPsv48B (NC_019266.1) and pPsv48C (NC_019292.2). On average, 95.87% (3.05 million reads) of wild‐type reads and 95.97% (3.28 million reads) of mutant reads were uniquely mapped (Figure S1).

A count matrix of all annotated genes in the Psv NCPPB 3335 genome across the six RNA‐seq samples was generated (Table S5). Count data were normalised using both edgeR, which employs trimmed mean of *M*‐values (TMM) (Robinson et al. 2010) and DESeq2 (median‐of‐ratios; Love et al. 2014), as required by DEgenes Hunter (Figure S2a). Principal component analysis (PCA) was then performed using MultiQC (Ewels et al. 2016), with samples clustering by strain: wild‐type replicates grouped together and were clearly separated from Δ*whpR* replicates along the principal component explaining the greatest variance (Figure S2b).

Differentially expressed genes (DEGs) were identified with DEgenes Hunter (Gayte et al. 2017), which calculates a combined *p*‐value through the Fisher method based on nominal *p*‐values provided by edgeR and DESeq2. Genes were considered differentially expressed when showing a fold change ≥ 2 (|log_2_FC| ≥ 1) and a false discovery rate (FDR)‐adjusted *p*‐value (Benjamini and Hochberg 1995) < 0.05 (equivalent to –log_10_(*p*) ≥ 1.3).

### 2.9 Real‐Time Quantitative PCR

RNA for real‐time quantitative PCR (RT‐qPCR) was extracted as described above. Complementary DNA (cDNA) was synthesised using the iScript cDNA Synthesis Kit (Bio‐Rad, CA, USA) with 1 μg of DNA‐free total RNA as a template. RT‐qPCR primers were designed in Primer3Plus (Untergasser et al. 2012) from target gene sequences. Primer specificity was validated via efficiency curve analysis (Vargas et al. 2011). Relative transcript levels were calculated by the ΔΔ cycle‐threshold (Ct) method (Livak and Schmittgen 2001), with normalisation to *gyrA* expression. ΔΔCt was defined as Ct (target gene) minus Ct (*gyrA*), and fold change was computed using the 2^−ΔΔCt^ formula (Pfaffl 2001). Each RT‐qPCR assay was run in technical triplicate, with three biological replicates. Relative expression was calculated by normalising the Δ*whpR* mutant to the wild‐type strain. Data were analysed in GraphPad Prism (v9.4.1), and group comparisons were made using ANOVA, with significance set at *p* < 0.05.

### 2.10 Plant Bioassays

Clonal micropropagated olive ( *Olea europaea* ) plants were derived from a single seedling germinated in vitro from cv. Arbequina. Micropropagation was carried out in Driver–Kuniyuki Walnut (DKW) medium (Driver and Kuniyuki 2022), following the protocol of Rodríguez‐Moreno et al. (2008) with minor modifications: explants were incubated for 7 weeks in a growth chamber at 25°C ± 1°C under a 16‐h photoperiod with a photon flux density of 60 μmol/m^2^/s, and rooted explants were transferred to hormone‐free DKW medium supplemented with 1 g/L activated charcoal and maintained under these conditions for at least 1 month prior to inoculation. Micropropagated (non‐woody) plants were inoculated following Caballo‐Ponce, Van Dillewijn, et al. (2017), with the following adjustments: bacterial suspensions were prepared in 10 mM MgCl₂ at an OD₆₀₀ of 0.5, corresponding to ~10^8^ colony‐forming units (CFU)/mL, and a single wound was made just above the petiole using a sterile needle, onto which 2 μL (~2 × 10^5^ CFU) were applied. Each experiment included six plants and was repeated three times. Symptom development was assessed at 30 days post‐inoculation using a stereomicroscope (Leica MZ FLIII; Leica Microsystems, Wetzlar, Germany).

To assess pathogenicity in woody plants, clonal in vitro olive plants were acclimatised to soil. Micropropagated plantlets were first transferred to jiffy pellets (Ling et al. 2022) and maintained for 2 weeks at 25°C± 1°C, 16 h light, 60% relative humidity and 300 ppm CO_2_, under transparent plastic covers. Plants were then transplanted into a soil mix: universal substrate (PROJAR S.A., Valencia, Spain), coconut fibre and vermiculite (10:10:1, v/v/v), supplemented with Osmocote Exact Standard, a slow‐release (3–4 month) fertiliser. Inoculations were performed on fully acclimatised woody olive plants grown for 3–6 months. Bacterial suspensions were prepared as above, and 20 μL (~2 × 10^6^ CFU) was introduced into wounds made with a sterile scalpel. Five independent plants per bacterial strain were inoculated, each at three distinct stem positions. Since knot size increases with proximity to the root, knot volume measurements were grouped by inoculation position. For each strain and position, the average volume of at least three individual knots was calculated, as previously described (Caballo‐Ponce, Van Dillewijn, et al. 2017; Moretti et al. 2019), and the overall knot volume per strain was then determined as the average of the three position‐specific means. Error bars indicate standard deviation. Statistical analyses were performed using one‐way ANOVA followed by Tukey's post hoc test (*α* = 0.05). Differences between strains were considered statistically significant at *p* < 0.05. Representative tumour images were recorded at the end of the experiment using a Nikon DXM 1200 camera.

Competitive index (CI) assays between wild‐type and mutant strains followed Caballo‐Ponce, Van Dillewijn, et al. (2017) and Matas et al. (2012). Suspensions were adjusted to an OD₆₀₀ of 0.5 (~10^8^ CFU/mL), mixed 1:1 and serially diluted. Inoculations involved 20 μL (~2 × 10^4^ CFU) on woody stems and 2 μL (~2 × 10^3^ CFU) on micropropagated (non‐woody) olive plants. At 30 days post‐inoculation (dpi) (non‐woody) and 100 dpi (woody plants), knots were harvested, macerated in 10 mM MgCl_2_ and plated on LB with Nf for Psv selection and Ch for fungal inhibition. Wild‐type colonies were distinguished from the Δ*whpR* mutant (Km^R^) by plating on LB agar ± Km. The CI was calculated as the mutant: wild‐type CFU ratio recovered from knots, normalised to the input ratio (confirmed to be approximately 1:1 by inoculum plating; Freter et al. 1981; Taylor et al. 1987).

## 3 Results

### 3.1 Bioinformatics Prediction of WHOP‐Encoded Enzymatic Functions Through Comparative Genomics

To gain insight into the unknown functions of the proteins encoded in the *dhoAB* and *ipoABC* operons, we first analysed their predicted roles using the KEGG pathways database. Figure 1 illustrates the organisation of WHOP genes in the Psv NCPPB 3335 genome. While no enzymatic function or pathway was assigned to WhpR, as expected for a regulatory protein, and IpoC, the remaining four proteins were primarily linked to the catabolism of synthetic fluorinated or chlorinated aromatic compounds, including fluorobenzoate, chlorobenzene and chlorocyclohexane, as well as the degradation of the petroleum by‐products toluene and styrene (Table S6). Given that these operons contribute to the adaptation of bacterial pathogens to woody hosts, an environment devoid of such anthropogenic compounds, it is unlikely that their actual functions correspond to those predicted by KEGG.

Next, to identify genomic sequences similar to the WHOP region of Psv NCPPB 3335 (WHOP‐like clusters) with potentially characterised functions in bacteria outside the *P. syringae* complex, we performed a MegaBLAST search using the entire 14,703‐nt WHOP region as the query. Except for *Pseudomonas* sp. DTU_2021, all hits with > 20% query coverage corresponded to *P. syringae* complex strains isolated from woody hosts. Then, we employed MultiGeneBlast to explore the distribution of *whpR* along with the *dhoAB* and *ipoABC* operons across available microbial genomes. In addition to the expected *P. syringae* complex sequences, the remaining hits were classified into 17 distinct WHOP‐like clusters according to their gene content, synteny and spatial separation of the operons. Most of these hits included other *Pseudomonas* species, along with representatives of the genera *Acinetobacter*, *Burkholderia* and *Marinobacterium*. These strains were predominantly sourced from plant‐related environments, such as agricultural soil or seeds, while a few strains were clinical isolates or sourced from waste or brackish water (Figure 1, Table S4).

Alongside *whpR* and the *dhoAB* and *ipoABC* operons, which were explicitly included in our search criteria, most WHOP‐like clusters also contain a complete *antABC* operon and the *antR* gene (Figure 1). Exceptions include *Pseudomonas* sp. B21‐028, *Pseudomonas* sp. DTU_2021, *Pseudomonas furukawaii*, all *Burkholderia* spp. and *Thauera* sp. K11. In contrast, the *catBCA* operon was restricted to *Pseudomonas oryzihabitans* , *Pseudomonas* sp. B21‐028 and most *Acinetobacte*r spp., with *Acinetobacter* sp. ADP1 (cluster 11) as an exception and *Pseudomonas* sp. KNUC1026 (cluster 6) specifically lacking *catA*. Spatial separation of operons was observed in *Acinetobacter* spp. (clusters 3 and 4), *Marinobacterium* spp. (cluster 12) and *Pseudomonas* sp. KNUC1026 (Figure 1, Table S4).

**FIGURE 2:**
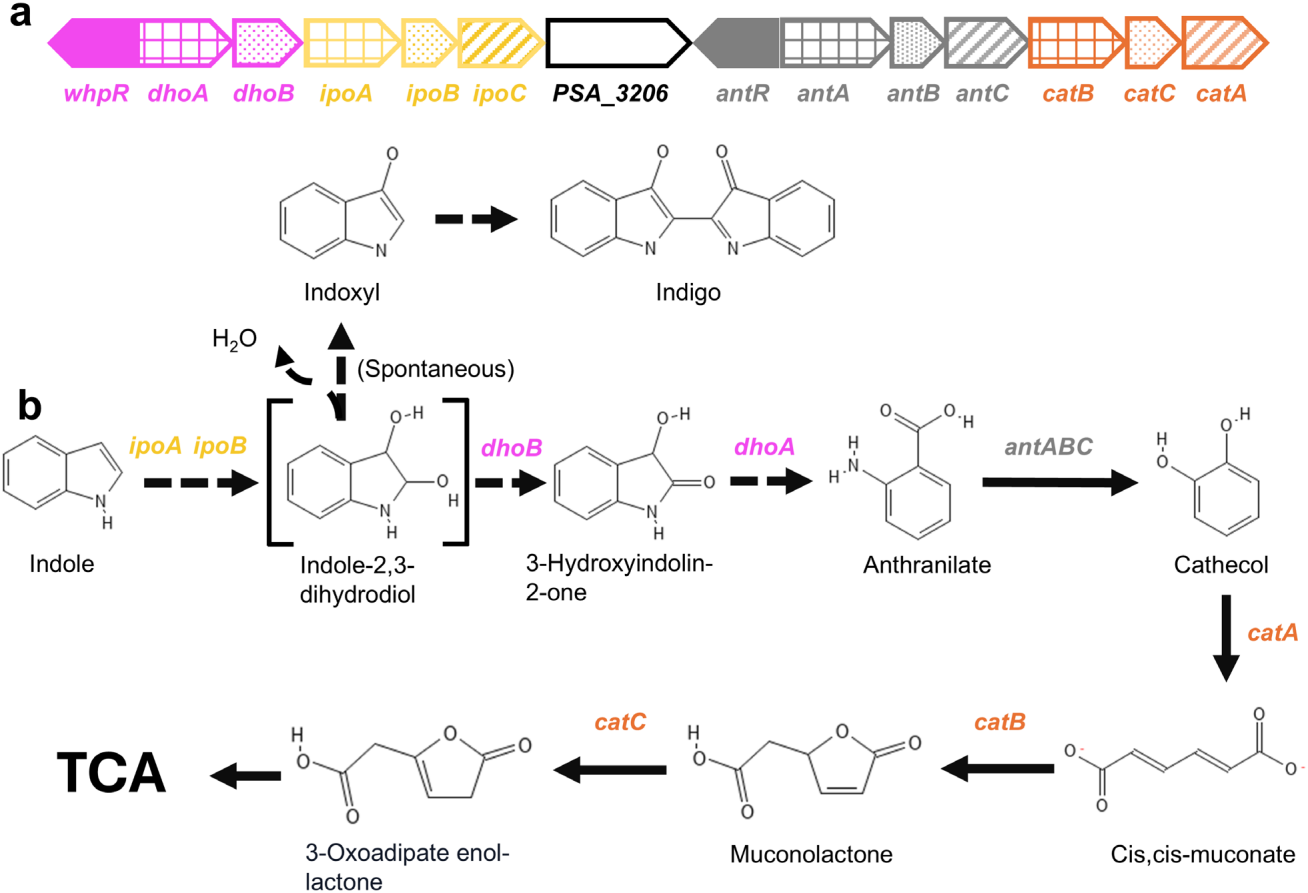
Proposed indole degradation pathway encoded by the WHOP region of *P. syringae* complex strains from woody hosts. (a) Organisation and distribution of WHOP genes in *P. savastanoi* pv. savastanoi NCPPB 3335. (b) Indole degradation pathway. The roles of the *dhoAB* and *ipoABC* operons, together with the spontaneous conversion of indole‐2,3‐dihydrodiol to indoxyl and indigo (dotted arrows), are based on pathways previously described (Sadauskas et al. 2017; Ma et al. 2018, 2019). The *ipoA* and *ipoB* genes encode a flavin‐dependent oxygenase system; *ipoC* an outer membrane channel of the MetA pathway, *dhoB* a short‐chain dehydrogenase, *dhoA* a cofactor‐independent oxygenase, and *whpR* an AraC/XylS‐type regulator. The downstream steps mediated by *antABC* and *catBCA* feed into the tricarboxylic acid (TCA) cycle and have been previously characterised (Caballo‐Ponce, Van Dillewijn, et al. 2017).

Bacteria from the genera *Acinetobacter* (Lin et al. 2015; Sadauskas et al. 2017) and *Burkholderia* (Kim et al. 2013; Ma et al. 2019) harbor genes homologous to *dho* and *ipo*, which have been designated *iif* (indole‐induced flavoprotein) and encode an indole degradation pathway. Specifically, in *Acinetobacter* sp. strain O153, isolated from the intestine of the crayfish *Orconectes limosus* , *iifC* and *iifD* (homologous to *ipoA* and *ipoB*, respectively) oxidise indole, forming indole‐2,3‐dihydrodiol. This unstable intermediate is further converted into anthranilate by *iifB* and *iifA* (homologous to *dhoB* and *dhoA*, respectively; Sadauskas et al. 2017). On the basis of these findings and our previous results on the *antABC* and *catBCA* operons (Caballo‐Ponce, Van Dillewijn, et al. 2017), we propose that the *dhoAB* and *ipoABC* operons of the *P. syringae* WHOP region mediate indole degradation via indole‐2,3‐dihydrodiol, leading to anthranilate. Anthranilate is converted into catechol by the *antABC* operon, which encodes the subunits and reductase component of anthranilate dioxygenase, and is then further metabolised by *catBCA* through the consecutive action of catechol 1,2‐dioxygenase, muconolactone isomerase and muconate cycloisomerase, channelling the intermediates into the β‐ketoadipate pathway (Figure 2).

The *whpR* gene was positioned at either the start or end of most clusters, except in *Pseudomonas* sp. B21‐028 and all *Acinetobacter* spp. in clusters 3 and 4. Its genomic context varied among clusters, with *whpR* flanking distinct operons. As in the WHOP region of Psv NCPPB 3335, it was located adjacent to *dhoAB* in clusters 8 and 12–17, near *ipoABC* in all *Acinetobacter* spp. and close to *antABC* (clusters 1, 5, 6, 9–11) or *catBCA* (cluster 2; Figure 1, Table S4). These findings suggest that the regulatory role of *whpR* may be shaped by its genomic context, potentially controlling distinct operons across indole‐degrading bacteria.

### 3.2 Domain and Structural Analysis of WhpR and IpoC

**FIGURE 3:**
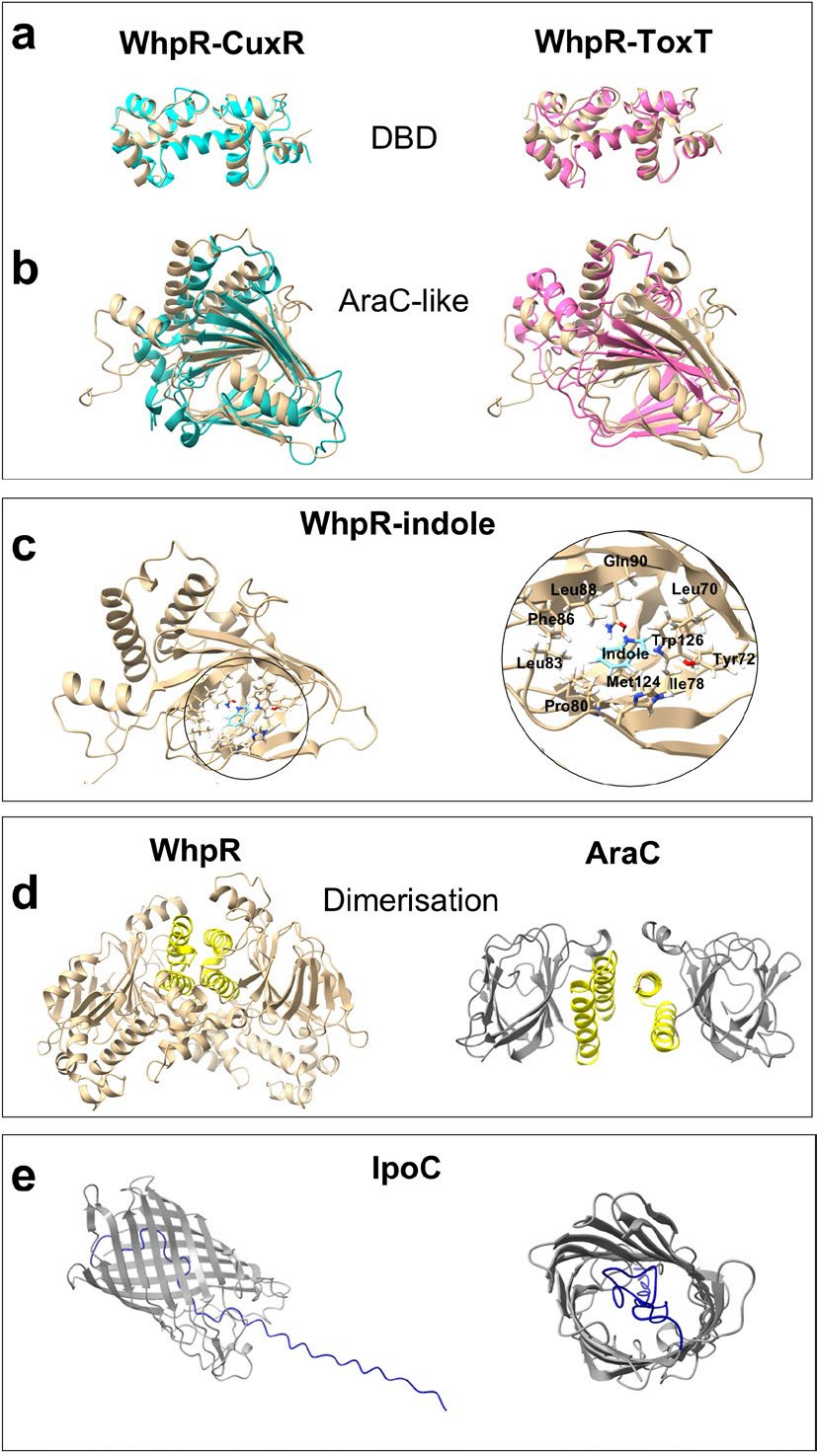
Structural analysis of WhpR and IpoC from *P. savastanoi* pv. savastanoi NCPPB 3335. (a, b) Superposition of the predicted AlphaFold structures of WhpR (light brown) with its homologues CuxR from *S. meliloti* (blue) and ToxT from *V. cholerae* (magenta), showing the DNA‐binding domain (DBD), featuring a helix–turn–helix (HTH) motif, and the AraC‐like ligand‐binding domain. (c) Docking analysis image of indole with the predicted 3D structure of WhpR using SwissDock 2 (left). The predicted ligand‐binding pocket (right) shows residues potentially stabilising indole through hydrophobic (Phe_86_, Leu_70_, Leu_88_, Ile_78_, Pro_80_, Met_124_), aromatic (Trp_126_, Tyr_72_) and polar (Gln_90_, His_42_) interactions. Residues are labelled with their three‐letter code and sequence position. (d) Predicted WhpR homodimer (left) compared with the crystallised AraC dimer (grey, right), both with α‐helical dimerisation interfaces highlighted in yellow (Soisson et al. 1997). (e) Side (left) and frontal (right) views of the predicted IpoC structure, highlighting the β‐barrel (grey) and a structurally undefined N‐terminal region (blue) including the predicted signal peptide (residues 1–21). All structures were visualised and edited using ChimeraX.

The potential function of the non‐enzymatic proteins, WhpR and IpoC, was inferred through structural analyses. InterPro analysis classified WhpR as a member of the AraC/XylS family of transcriptional regulators (IPR050204), with an AraC ligand‐binding‐like domain (residues 32–209) and a helix–turn–helix (HTH) DNA‐binding domain (DBD) (residues 236–335). Using I‐TASSER, two structural homologues of WhpR were identified: CuxR, a transcriptional activator from *Sinorhizobium meliloti* involved in exopolysaccharide biosynthesis under elevated c‐di‐GMP levels (Schäper et al. 2017), and ToxT, a regulator of cholera toxin and toxin‐coregulated pilus expression in *Vibrio cholerae* (Lowden et al. 2010). Despite sharing only 16% and 8% sequence identity with WhpR, respectively, CuxR and ToxT exhibited strong structural similarity. Superposition of the AlphaFold‐predicted structure of WhpR with that of ToxT, or with the crystallised structure of CuxR, revealed close resemblance in both the DBD and the AraC‐like domain of WhpR compared with these homologues (Figure 3). At the DBD (Figure 3a), pruned Root Mean Square Deviation (RMSD) values, representing the average distance between equivalent atoms after excluding outliers, indicated very high similarity, with WhpR–CuxR showing slightly lower structural deviation (1.106 Å over 88 atom pairs) than WhpR–ToxT (1.115 Å over 49 atom pairs). In the AraC‐like domain (Figure 3b), alignment with CuxR again showed lower pruned RMSD (1.027 Å over 48 atom pairs) than with ToxT (1.136 Å over 32 atom pairs), reinforcing the closer structural similarity between WhpR and CuxR.

SwissDock 2 docking analyses of WhpR with indole, anthranilate and taurine as a negative control predicted a stable binding mode for anthranilate (top cluster: 7 poses; best ΔG −6.25 kcal/mol; mean ΔG −6.15 kcal/mol), while indole showed slightly more variable binding (top cluster: 4 poses; best ΔG −6.34 kcal/mol; mean ΔG −5.87 kcal/mol). The predicted indole‐binding pocket was stabilised primarily by hydrophobic and aromatic amino acid contacts, with additional polar contributions (Figure 3c). Taurine, in contrast, produced a dispersed docking pattern (top cluster: 3/50), consistent with nonspecific binding. These results support indole and anthranilate as WhpR ligands and indicate a defined pocket within its AraC‐like domain. In addition, AlphaFold predicted that WhpR dimerises through the same α‐helical interface described for AraC by Soisson et al. (1997) and later shown to be stabilised by c‐di‐GMP in CuxR (Schäper et al. 2017; Figure 3d). These results suggest a conserved dimerisation strategy within this transcriptional regulator family.

The *ipoC* gene, present in all WHOP‐like clusters except *Thauera* sp. K11 (Figure 1, Table S4), is orthologous to *iifE* in *Acinetobacter* sp. O153, which encodes an outer membrane channel with an uncharacterised role (Sadauskas et al. 2017). InterPro analysis identified IpoC from Psv NCPPB 3335 as a putative member of the β‐barrel porin/alpha‐amylase or MetA‐pathway of the phenol degradation superfamily (IPR025737). It contains a signal peptide region (residues 1–21) and an extracellular region (residues 22–311), predicted to be part of a membrane‐bound protein. AlphaFold analysis further suggested that this extracellular region adopts a β‐barrel structure, forming a channel (Figure 3e).

### 3.3 *ipoABC* Operon Mediates Indole Utilisation and Cell Aggregation in *P. savastanoi*

**FIGURE 4:**
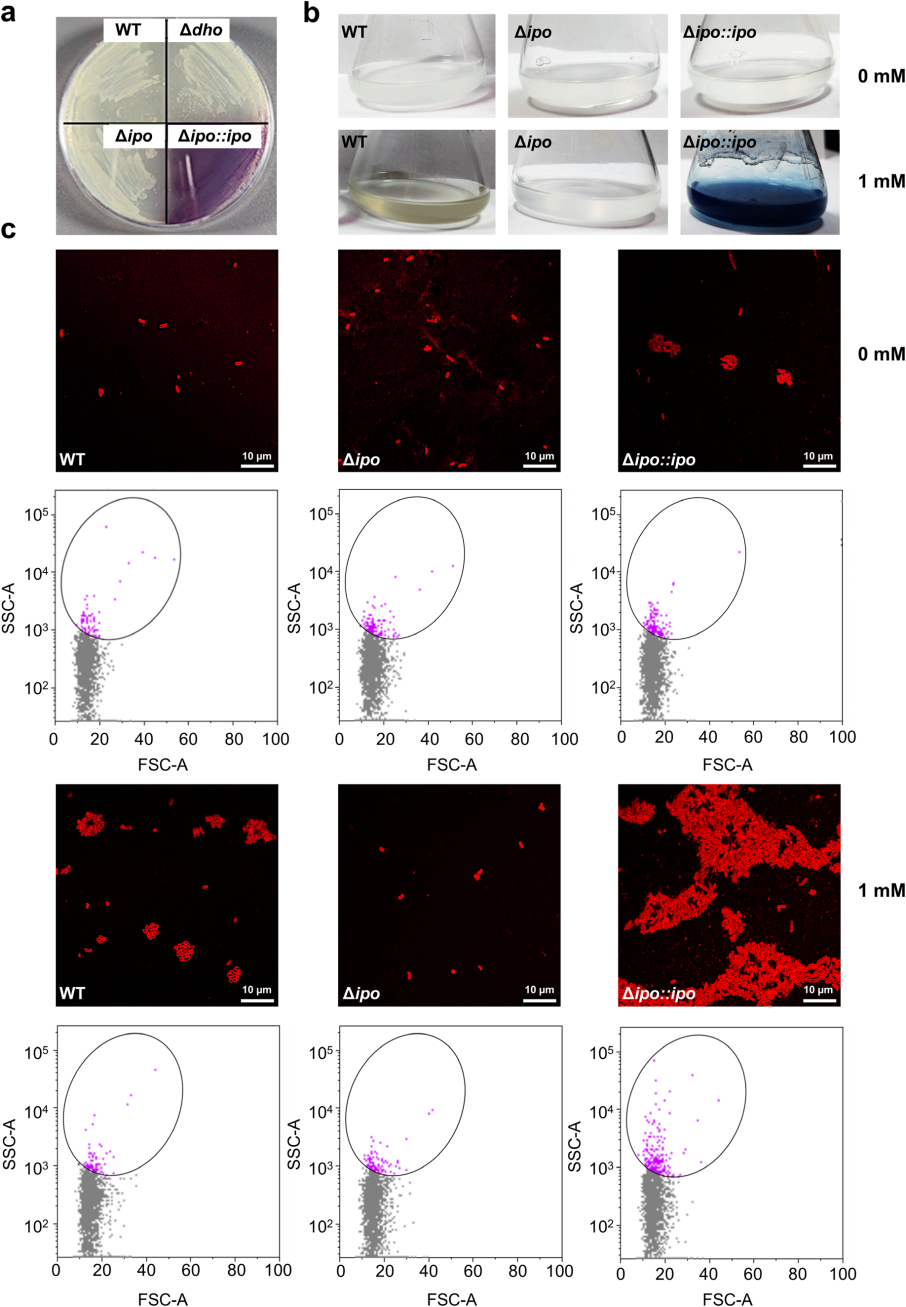
The *ipoABC* operon mediates indole degradation, indigo production and cell aggregation in *P. savastanoi* pv. savastanoi NCPPB 3335. (a) Indole degradation on LB agar with 5‐bromoindoline for WT (wild‐type NCPPB 3335), Δ*dhoAB*, Δ*ipoABC* and the complemented (Δ*ipo::ipo*) strains. (b) Cultures grown in M9 medium without (upper) or with 1 mM indole (lower) for 24 h. (c) Confocal images of FM4‐64‐stained cultures from (b), with flow cytometry plots of unstained samples below, showing side scatter area (SSC‐A) versus forward scatter area (FSC‐A). An elliptical gate (magenta) defines a common population across plots.

The role of the *ipoABC* and *dhoAB* operons in indole metabolism was first investigated using a 5‐bromoindoline plate assay, in which this indole analogue generates a purple compound upon degradation (Sadauskas et al. 2017). The assay included wild‐type Psv NCPPB 3335, its Δ*dhoAB* and Δ*ipoABC* mutants and a complemented Δ*ipoABC* strain (Δ*ipo::ipo*), which expresses the *ipoABC* operon from its native promoter on a plasmid. Only the Δ*ipo::ipo* strain produced the characteristic purple colouration, indicating 5‐bromoindoline metabolism dependent on *ipoABC* activity. No visible pigment formation was observed for the wild type, Δ*dhoAB*, or Δ*ipoABC* strains (Figure 4a).

To further assess indole transformation, Psv strains were incubated for 24 h in M9 minimal medium supplemented with 1 mM indole. Cultures of the wild‐type strain developed a visible yellowish colouration, whereas the Δ*ipoABC* mutant, as well as all strains grown in the absence of indole, remained colourless. In contrast, the Δ*ipo::ipo* strain exhibited a striking blue pigmentation and accumulated an insoluble precipitate, consistent with indigo production driven by multicopy plasmid‐mediated *ipoABC* overexpression (Figure 4b). These results agree with our previous study, in which overexpression of the *ipoABC* operon in either *E. coli* or the Δ*ipoABC* mutant background led to the formation of a blue pigment later confirmed as indigo by high‐performance liquid chromatography (HPLC) analysis (Caballo‐Ponce, Van Dillewijn, et al. 2017). Meanwhile, the yellow colouration observed in the wild‐type culture, absent in the Δ*ipoABC* mutant, is consistent with the formation of coloured by‐products reported in other bacteria during aerobic indole degradation involving *ipo* gene homologues (Qu et al. 2015; Kim et al. 2016).

In addition to pigment production, a distinctive blue ring, apparently composed of cell aggregates attached to the inner flask wall, formed after 24 h of incubation in the indole‐amended culture of the Δ*ipo::ipo* strain. This structure was not observed in cultures of any other strain, regardless of indole presence (Figure 4b). Crystal violet staining revealed retention only in this culture (data not shown), indicating the formation of biofilm‐like cell aggregates specifically linked to *ipoABC* overexpression in the presence of indole. To further investigate potential cell aggregation in the liquid cultures, bacterial cells were stained with FM4‐64, a lipophilic fluorescent dye that labels the outer membrane, and visualised by confocal fluorescence microscopy. In the absence of indole, all strains showed typical rod‐shaped morphology. Most cells appeared as individuals in the wild‐type and Δ*ipoABC* cultures, while the Δ*ipo::ipo* strain frequently formed small clumps. In the presence of indole, wild‐type and Δ*ipoABC* cells became smaller and more spherical, with the wild type also forming small aggregates. In contrast, Δ*ipo::ipo* cells retained their rod shape and formed large, compact aggregates composed of densely packed cells, suggesting strong intercellular adhesion (Figure 4c). Flow cytometry analysis of cell size and complexity supported the observed biofilm and aggregation phenotypes. In the presence of indole, a subset of the Δ*ipo::ipo* population exhibited increased side scatter area (SSC‐A) compared to the Δ*ipoABC* mutant and wild‐type strains (Figure 4c), consistent with higher cellular complexity or aggregation. Together, these results show that indole‐induced cell aggregation and biofilm formation also depend on the *ipoABC* operon.

### 3.4 WhpR Regulates Indole Catabolism Genes Within and Beyond the WHOP Region

AraC/XylS family regulators control either single operons or coordinate diverse virulence‐ or stress‐related functions encoded at distant genomic loci (Gallegos et al. 1997). This regulatory versatility, combined with the variable genomic context of *whpR* across different bacteria (Figure 1), suggested that WhpR might regulate gene expression both within the WHOP region and at other genomic loci in *P. syringae* pathogens of woody hosts. To investigate this, RNA was purified from Psv NCPPB 3335 and its Δ*whpR* mutant after 6 h of induction in HIM medium (Huynh et al. 1989). Three RNA‐seq libraries were generated per strain, and over 95% of the reads from both the wild‐type and mutant samples were uniquely mapped (Figure S1). PCA showed that wild‐type replicates clustered together and clearly separated from Δ*whpR* replicates along the principal component (Figure S2).

**FIGURE 5:**
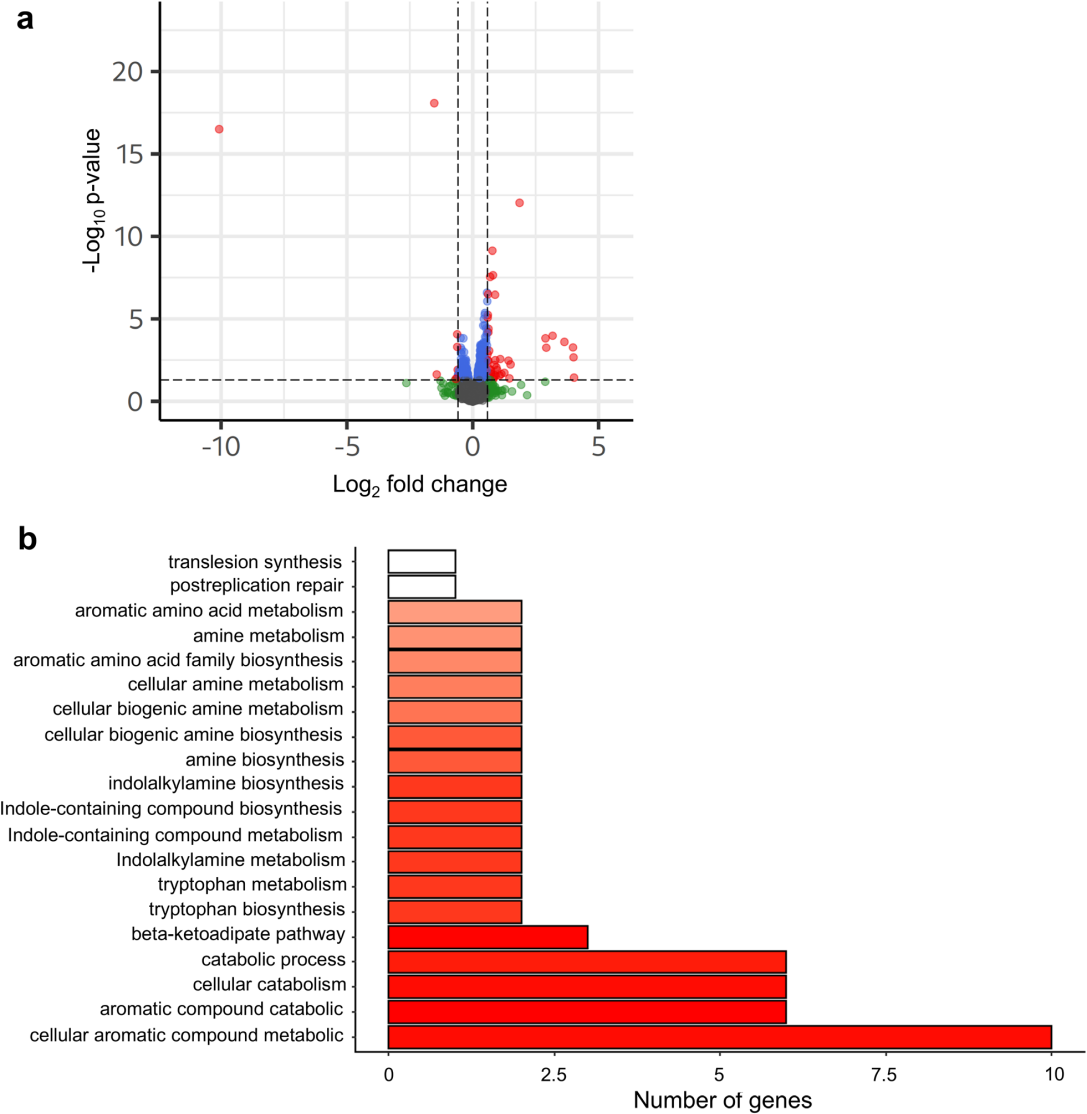
Identification of the WhpR regulon in *P. savastanoi* pv. savastanoi NCPPB 3335 by RNA‐Seq. (a) Volcano plot of differentially expressed genes (DEGs) in the △*whpR* mutant versus wild‐type NCPPB 3335 (see Table S7). Horizontal and vertical dashed lines indicate the *p*‐value (−log_10_(*p*) ≥ 1.3) and fold change (|log_2_FC| ≥ 1) thresholds, respectively. Genes are colour‐coded: Red, DEGs meeting both thresholds; grey, neither threshold; green, exceeding fold threshold only; blue, exceeding *p*‐value threshold only. (b) Gene Ontology (GO) functional enrichment analysis of DEGs. The intensity of red bars reflects the number of genes in each GO category.

**FIGURE 6:**
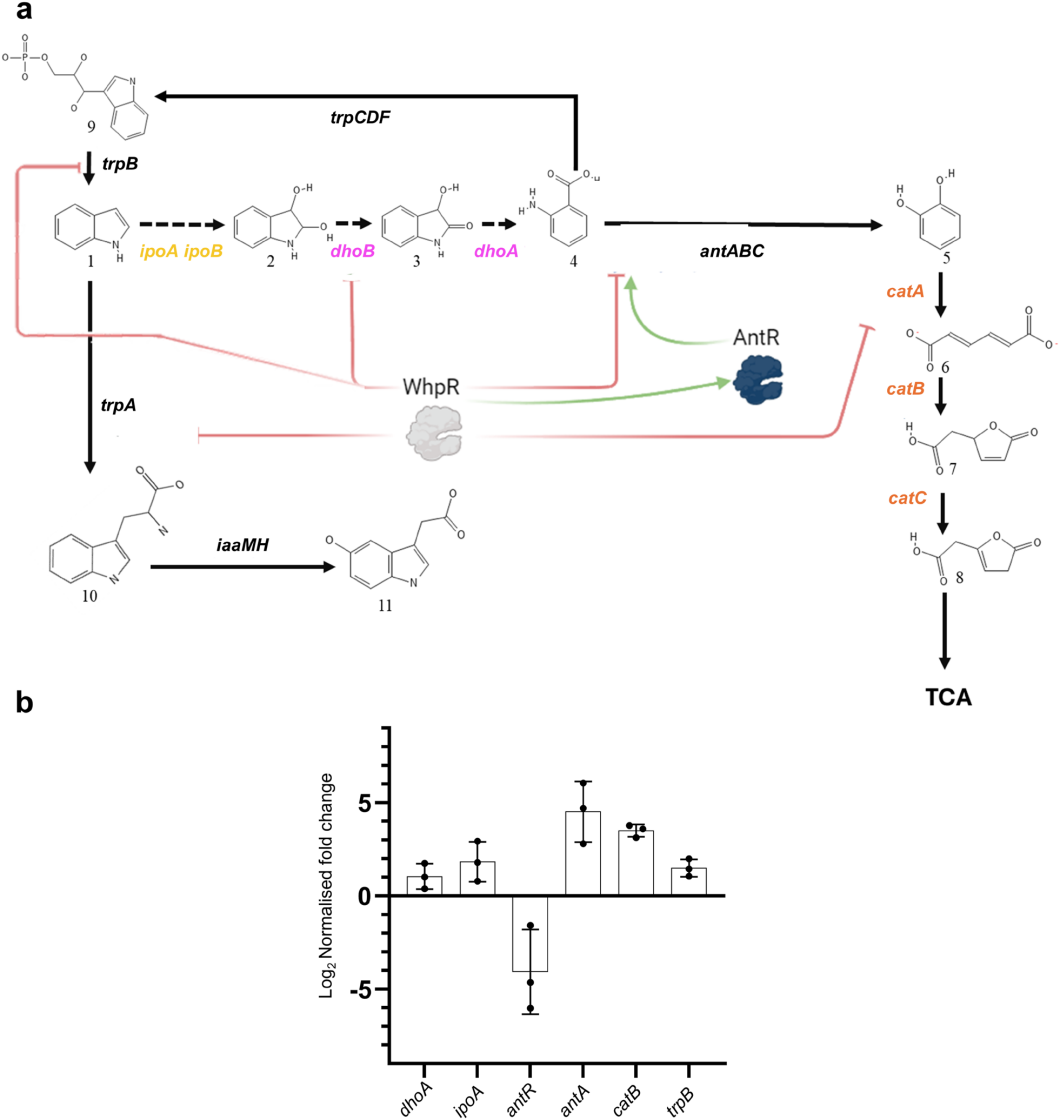
WhpR‐regulated indole metabolism in *P. savastanoi* pv. savastanoi NCPPB 3335. (a) Metabolic pathway showing (1) indole, (2) indole‐2,3‐dihydrodiol, (3) 3‐hydroxyindolin‐2‐one, (4) anthranilate, (5) catechol, (6) cis,cis‐muconate, (7) muconolactone, (8) 3‐oxoadipate enol‐lactone, (9) indoleglycerol phosphate, (10) L‐tryptophan and TCA (tricarboxylic acid cycle). Dotted arrows indicate proposed enzymatic steps based on pathways previously described (Sadauskas et al. 2017; Ma et al. 2018, 2019). Green arrows and red T‐bars denote transcriptional activation and repression by WhpR, respectively. (b) RT‐qPCR of selected WHOP genes in Δ*whpR* versus wild‐type 6 h after transfer to Hrp‐Inducing Medium (HIM), normalised to *gyrA*. Data represent three independent experiments ± SD; significance by ANOVA, *p* < 0.05.

RNA‐seq analysis identified 49 DEGs in the Δ*whpR* mutant relative to the wild‐type strain, with a total of 42 upregulated and seven downregulated genes (Figure 5a, Table S7). Functional enrichment analysis showed that most DEGs were involved in the metabolism of aromatic compounds (Figure 5b). Several genes encoded in the Psv NCPPB 3335 WHOP region were upregulated in the Δ*whpR* mutant, with particularly strong expression observed for the *dhoAB*, *antABC* and *catABC* operons. Fold change values within these operons ranged from 3.6‐fold to 16.0‐fold increases in gene expression, with the highest values in *antABC* genes. In contrast, the regulatory gene *antR* was downregulated. Additionally, the *trpAB* operon, located outside the WHOP region and encoding the *α* and *β* subunits of tryptophan synthase, was also upregulated in the Δ*whpR* mutant (Tables S6 and S7). These results show that WhpR negatively regulates transcription of most operons within the WHOP region, as well as the metabolically related *trpAB* operon. The upregulation of all these indole‐related genes, further confirmed by RT‐qPCR analysis, and their associated metabolic pathways are illustrated in Figure 6. Given the role of the *trpAB* operon in the biosynthesis of the auxin precursor L‐tryptophan, we also quantified IAA levels in the culture supernatants of the wild‐type and Δ*whpR* strains; however, no significant differences were observed under the tested conditions (data not shown).

In the Δ*whpR* mutant, 39 genes located outside the WHOP region exhibited differential expression compared to the wild‐type strain, with 32 genes upregulated and six downregulated. The upregulated genes are involved in polysaccharide, sulphite and undefined compound transport, toxin‐antitoxin mechanisms, osmotic stress response and recombination. Over one‐third of these genes are annotated as hypothetical proteins or encode domains of unknown function. The downregulated genes were primarily associated with transport, regulation of transcription and the metabolism of amino acids and pyrimidines (Table S7).

### 3.5 WhpR Modulates Virulence and Fitness of Psv NCPPB 3335 in Woody Olive Plants

**FIGURE 7:**
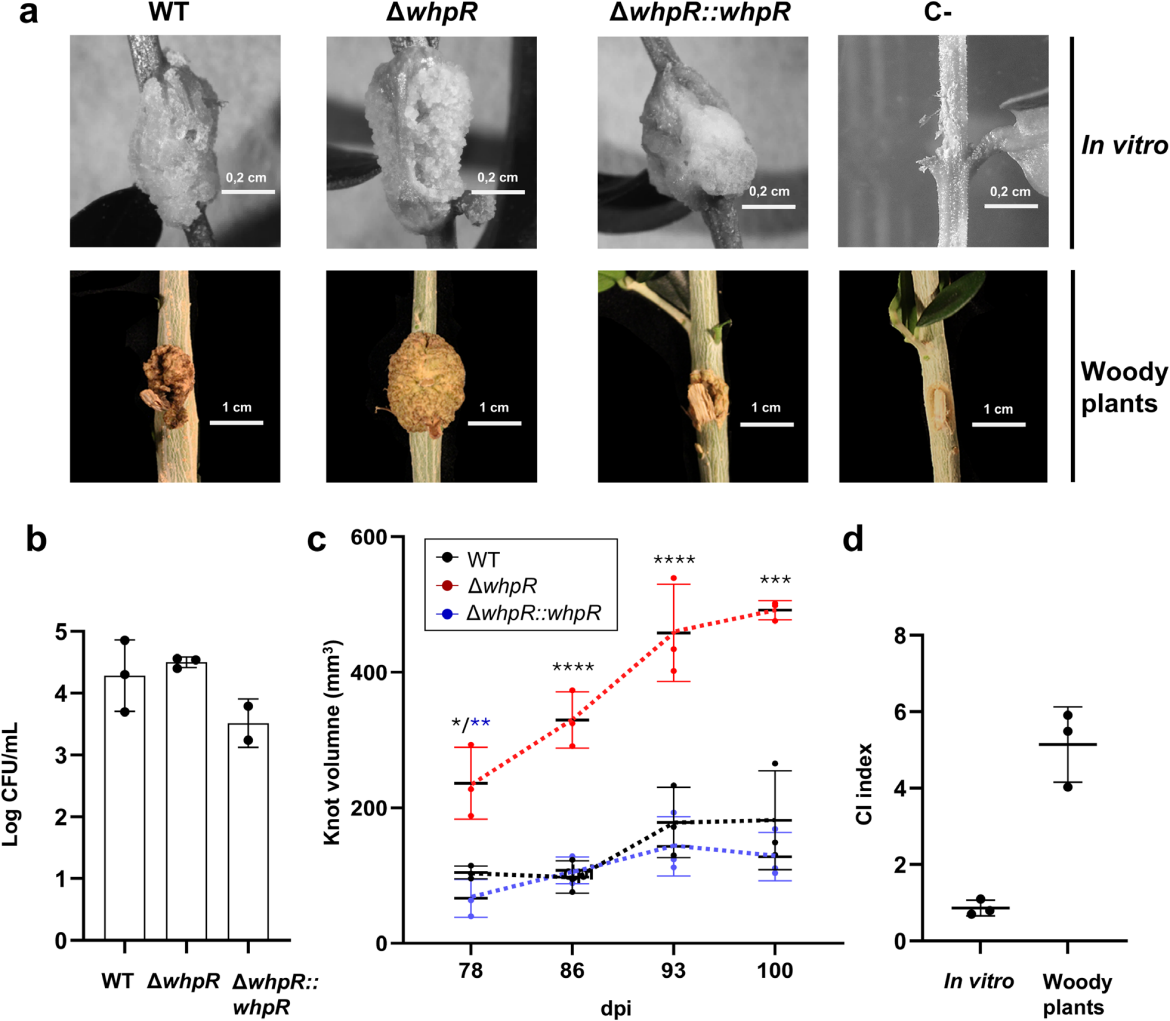
Role of WhpR in the virulence of *P. savastanoi* pv. savastanoi NCPPB 3335 in olive plants. (a) Knots induced by the wild‐type (WT), Δ*whpR*, and the complemented (*ΔwhpR::whpR*) strains in non‐woody micropropagated plants at 30 days post‐inoculation (dpi) (upper) and woody plants at 100 dpi (lower). Negative control: 10 mM MgCl_2_. (b) Bacterial populations (log CFU/mL) in non‐woody plants at 30 dpi; error bars indicate the standard error of the mean. (c) Knot volumes in woody olive plants at the indicated time points. Values represent means of position‐specific averages across three stem heights; error bars indicate standard deviation. One‐way ANOVA with Tukey's post hoc test (*α* = 0.05); asterisks indicate significant differences (*p* < 0.05) of Δ*whpR* versus WT (*) and Δ*whpR::whpR* (**). (d) Competitive index (CI) from mixed inoculations of WT and Δ*whpR* in non‐woody and woody olive plants. Data represent the means of three biological replicates (*n* = 3), with error bars indicating the standard error of the mean. Statistical significance was determined using a two‐tailed Student's *t* test (*p* < 0.05).

Given that most operons encoded in the WHOP region and negatively regulated by WhpR (Figure 6, Table S7) are critical for full virulence of Psv NCPPB 3335 in woody olive plants (Caballo‐Ponce, Van Dillewijn, et al. 2017), we investigated the role of WhpR in the virulence of this pathogen. For this purpose, virulence assays were performed in two different model systems, micropropagated olive explants (non‐woody plants) and three‐month‐old woody olive plants. Plants were inoculated with either the wild‐type Psv NCPPB 3335 strain, its Δ*whpR* mutant, or the complemented strain Δ*whpR::whpR*. At 30 dpi, no significant differences in tumour size were observed in micropropagated plants among the strains tested (Figure 7a). Similarly, all three strains reached equal populations within the tumours (approximately 10^4^–10^5^ CFU per knot) (Figure 7b). A competition assay between the wild‐type strain and the Δ*whpR* mutant yielded a competitive index (CI) not significantly different from 1.0, further confirming that both strains are equally competitive in non‐woody plants.

In contrast, tumour volumes induced in woody olive plants by the Δ*whpR* mutant were significantly larger than those of the wild‐type and the complemented strains from 78 to 100 dpi (Figure 7c). In addition, a CI near 5.0 was obtained at 100 dpi in a competition assay between the Δ*whpR* mutant and the wild‐type strain, demonstrating an enhanced fitness of the Δ*whpR* mutant during woody plant infection (Figure 7d).

## 4 Discussion

The WHOP region of *P. syringae* pathogens of woody hosts represents a distinctive genetic module whose ecological and functional roles have remained only partially understood. Traditionally viewed as a conserved module restricted to these strains, this perception was based largely on conventional BLAST searches, which often overlook homologous gene clusters that have undergone rearrangement or do not maintain strict synteny. In this study, we combined comparative genomics with MultiGeneBlast, a more versatile tool that identifies gene clusters based on protein homology and organisation, to explore the broader distribution of genes homologous to the WHOP region. This approach revealed analogous gene clusters (WHOP‐like clusters) not only within the *P. syringae* complex but also in other *Pseudomonas* species and in genera such as *Acinetobacter*, *Burkholderia* and *Marinobacterium*. Strains that conserve the *whpR* gene alongside most genes encoded in the *P. syringae* WHOP region are predominantly associated with plant‐related or environmental sources, including agricultural soils, seeds and rhizospheres (Figure 1, Table S4). These findings expand the known taxonomic and ecological range of pathways related to those encoded in the WHOP region and support a conserved role in phenolic compound degradation, potentially contributing to bacterial adaptation in complex plant‐associated niches.

Beyond the *P. syringae* complex, WHOP‐like clusters retaining the *whpR* (*iifR*) gene together with the *iifABCDE* operon were also identified in a limited number of *Pseudomonas* strains and in several *Burkholderia* isolates, which originate from a broader range of environments, including both clinical and environmental sources (Figure 1, Table S4). Homologues of these genes have also been functionally characterised in diverse bacterial species from clinical, environmental and aquatic sources (Lin et al. 2015; Sadauskas et al. 2017; Ma et al. 2018, 2019), and some studies reported that *P. syringae* pv. actinidiae carries orthologues of all five *iif* genes (Lin et al. 2015; Sadauskas et al. 2017). In most of these cases, orthologues of *dhoAB* and *ipoABC*, which together constitute the *iifABCDEF* operon, have been shown to mediate the degradation of indole into anthranilate. On the basis of these findings, we propose that the *dhoAB* and *ipoABC* operons of the WHOP region perform a conserved role in this transformation (Figure 2), with IpoC specifically predicted to function as a membrane‐bound protein with a β‐barrel domain characteristic of porins and associated with the phenol degradation superfamily (Figure 3d). Its presence in WHOP‐like clusters suggests a role in exporting indole‐derived intermediates, potentially preventing toxic intracellular accumulation and facilitating adaptation to indole‐ or phenol‐rich environments. Experimental validation, such as membrane localisation studies or transport assays with indole‐derived metabolites, would be necessary to substantiate this proposed function. Indole and its derivatives are ubiquitous in nature, serving as signalling molecules in prokaryotes and exerting broader physiological functions in eukaryotes. As indole can inhibit bacterial growth, many bacteria have evolved enzymatic strategies to mitigate its toxicity by converting it into less harmful compounds (Lee et al. 2015; Kumar et al. 2021). Accordingly, the presence of *dhoAB* and *ipoABC* homologues in phylogenetically and ecologically diverse bacteria underscores the broader relevance of indole degradation beyond plant‐associated contexts.

Our previous work demonstrated that overexpression of the *ipoABC* operon in either *E. coli* or a *ΔipoABC* mutant of Psv NCPPB 3335 leads to the formation of a blue pigment, later confirmed as indigo (Caballo‐Ponce, Van Dillewijn, et al. 2017). This process was initially interpreted according to the pathway proposed by Ensley et al. (1983), in which indole is oxidised by naphthalene dioxygenase to cis‐indole‐2,3‐dihydrodiol, followed by spontaneous conversion to indoxyl and dimerisation into indigo. However, our current results, supported by recent findings in *Acinetobacter* sp. O153, suggest that indole degradation in Psv follows a different route (Figure 2). In this pathway, indole is first oxidised to indole‐2,3‐dihydrodiol, an unstable intermediate that can spontaneously dehydrate to form indoxyl and indigo, unless diverted by IifB (DhoB) and IifA (DhoA) to produce anthranilate (Sadauskas et al. 2017; Ma et al. 2018, 2019). Consistently, indigo formation in Psv was observed only in the strain overproducing *ipoABC*, while the wild‐type strain accumulated a yellow pigment in the presence of indole.

The yellow colouration observed in wild‐type Psv cultures may reflect the accumulation of alternative degradation products, such as isatin or related oxidised intermediates. Several bacterial species have been reported to produce isatin or yellow indole‐derived compounds under aerobic conditions. In *Acinetobacter* sp. O153, isatin was suggested to arise as a dead‐end product of spontaneous oxidation (Sadauskas et al. 2017). Other examples include *Alcaligenes* and *Arthrobacter* strains (Kim et al. 2016); *Cupriavidus* sp. SHE, which accumulates a yellow metabolite (C_15_H_8_N_2_O_3_), consistent with an indole oxidation product (Qu et al. 2015); and *Burkholderia* sp. IDO3, which produces a range of intermediates, including indole‐2,3‐dihydrodiol, 3‐hydroxyindolin‐2‐one, isatin and isatinate, ultimately yielding anthranilate (Ma et al. 2019).

In Psv, indole exposure induced aggregation and biofilm formation in an *ipoABC*‐dependent manner, with these phenotypes most pronounced in the Δ*ipo::ipo* strain (Figure 4). This suggests that indole‐derived products or intermediates generated through the *ipoABC*‐mediated pathway may trigger these multicellular behaviours. Indole is increasingly recognised as a signalling molecule that regulates diverse bacterial traits, including motility, multicellularity, stress responses and biofilm development (Di Martino et al. 2003; Hu et al. 2010; Lee et al. 2015). However, the effects of indole can vary across species and strains. In *Agrobacterium tumefaciens* , indole reduces motility while enhancing biofilm formation and antibiotic tolerance (Lee et al. 2015). In *Pantoea agglomerans* , it promotes the formation of multicellular aggregates but inhibits biofilm development (Yu et al. 2016). In contrast, *Arthrobacter* and *Alcaligenes* strains respond to indole by reducing their cell size as an adaptive mechanism (Kim et al. 2016). Our structural analyses identified WhpR as a member of the AraC family of transcriptional regulators (Figure 3), suggesting that it acts as a ligand‐responsive regulator. AraC‐type proteins are widely conserved and often modulate key cellular functions in response to environmental or intracellular signals (Gallegos et al. 1997). The structural similarity of WhpR to ToxT in *V. cholerae* (Lowden et al. 2010) and CuxR in *Sinorhizobium* (Schäper et al. 2017), both involved in host interaction, supports its potential role in integrating external cues to modulate gene expression. RNA‐seq analysis confirmed that WhpR represses all operons within the Psv NCPPB 3335 WHOP region (except *ipoABC*) as well as *trpAB*, located elsewhere in the genome and also involved in indole metabolism. As shown in Figure 6, anthranilate produced by the enzymes encoded in *dhoAB* can potentially be converted into indole‐3‐glycerol phosphate via *trpCDE* and subsequently into indole and tryptophan by *trpB* and *trpA*. This coordinated regulation may optimise resource use under conditions of indole accumulation. Moreover, WhpR also regulates other transcriptional regulators, such as *antR* and a gene encoding a FecR family protein (Table S7), reflecting a hierarchical control mechanism described for other AraC/XylS‐type proteins (Gallegos et al. 1997), including ToxT (Yu and Dirita 1999).

In addition to repressing WHOP operons, WhpR influences the expression of multiple genes located elsewhere in the genome, including those involved in transport, toxin‐antitoxin systems, osmotic stress response and recombination (Table S7). Although not all these genes are directly linked to indole metabolism, their co‐regulation suggests that WhpR may coordinate a broader cellular response to stress or environmental cues. In line with the ability of AraC‐family regulators to respond to small molecule ligands (Gallegos et al. 1997), SwissDock 2 docking analyses suggest that WhpR interacts with indole and anthranilate (Figure 3c), indicating that its regulatory activity may be modulated by indole compounds. Nevertheless, this hypothesis requires experimental validation, such as electrophoretic mobility shift assays (EMSA) in the presence of indole or related intermediates. For instance, the binding of cis‐palmitoleic acid to ToxT prevents its DNA association, reducing virulence gene expression in *V. cholerae* (Lowden et al. 2010), whereas c‐di‐GMP binding promotes dimerisation and DNA binding of CuxR in *Sinorhizobium* (Schäper et al. 2017). Taken together, these findings support the idea that WhpR functions as a global regulator integrating indole‐dependent signals, potentially enhancing *P. syringae* survival under stress or host‐associated conditions.

Although WhpR represses the *trpAB* operon, in vitro IAA levels were not significantly affected in the Δ*whpR* mutant compared to the wild‐type strain, in either minimal or apoplast‐mimicking media (data not shown). The increased tumour volumes observed in woody olive plants infected with the mutant (Figure 7) raise the possibility that IAA levels might still differ *in planta*. The absence of differences in vitro does not exclude in vivo modulation, as plant‐derived cues could alter IAA biosynthesis or its effects during infection. It is also possible that differences in IAA production could be detected under different growth conditions. Alternatively, the upregulation of *trpAB* in the Δ*whpR* mutant might be redirected towards other tryptophan‐derived metabolites, such as indol‐3‐acetaldehyde, indol‐3‐acetonitrile and tryptamine, which have been detected in other members of the *P. syringae* complex (Kunkel and Harper 2018) or could be subject to post‐transcriptional regulation that limits IAA production. Moreover, tumour development in woody tissues involves additional virulence‐related pathways and plant responses (Caballo‐Ponce, Murillo, et al. 2017), which may be influenced by WhpR‐regulated genes outside the WHOP region.

The hypervirulent phenotype of the Δ*whpR* mutant in woody olive plants, but not in non‐woody explants, agrees with previous findings that WHOP genes are specifically required for infection of woody hosts (Caballo‐Ponce, Van Dillewijn, et al. 2017). This pattern suggests that WhpR‐regulated pathways support the detoxification or degradation of plant‐derived indolic compounds that are less abundant in herbaceous tissues. Both woody and herbaceous plants produce indole‐containing defence metabolites, including phytoalexins, diverse tryptophan‐derived compounds and the phenolic phytohormone salicylic acid (SA) (Agrios 2005; Tiku 2020). Systemic acquired resistance (SAR) triggered by *P. syringae* induces more than 20 indolic metabolites, with camalexin, indol‐3‐ylmethylamine and indole‐3‐carboxylic acid being the major compounds accumulating at infection sites. While indole‐3‐carbaldehyde accumulates in both infected and distal leaves, camalexin production remains confined to local infection sites, and systemically elevated indoles are dispensable for the associated rise in SA, the central phytohormone of SAR signalling (Stahl et al. 2016). Thus, SA functions as the systemic signal, whereas indolic metabolites exert stronger localised effects on pathogen colonisation. In woody tissues, the metabolic environment is further shaped by lignin and lignans, abundant aromatic compounds with antibacterial activity (Li et al. 2023; Vinchira‐Villarraga et al. 2024). Together, these indolic and aromatic metabolites may impose unique chemical constraints on bacterial colonisers. The coordinated regulation of WHOP‐encoded enzymes and tryptophan‐related pathways by WhpR may facilitate detoxification or catabolism of such molecules. Collectively, our findings support a model in which WHOP and WhpR integrate environmental sensing with indole metabolism to modulate bacterial fitness and virulence in woody host interactions. This model further implies that *P. syringae* pathogens of woody hosts face a dual indolic environment, encountering both plant‐derived defence metabolites and its own microbially produced indole via tryptophan catabolism, with WhpR acting as a central regulator to balance detoxification and metabolic adaptation.

## 5 Conclusions

Bacterial diseases of woody plants, including major fruit crops, involve infection strategies distinct from those of herbaceous plants, with most of the underlying molecular mechanisms yet to be fully elucidated. The WHOP genomic island of *P. syringae* has emerged as a fundamental contributor to virulence in woody plant pathogens. Despite the recognised importance and established role of this region in degrading aromatic compounds such as anthranilate and catechol, the full extent of its metabolic functions, regulatory dynamics and contributions to host adaptation remain unresolved. In this study, we fill a key gap in the metabolic model by demonstrating that the WHOP region mediates indole degradation and likely contributes to anthranilate formation, thereby integrating indole detoxification into a broader aromatic catabolic network. We also define the WhpR regulon as a central regulatory hub linking this pathway with additional genes involved in indole metabolism, including tryptophan biosynthesis. Beyond its metabolic functions, the WHOP region orchestrates bacterial lifestyle transitions by connecting indole metabolism to multicellular behaviours such as biofilm formation and cell aggregation. The regulatory role of WhpR fine‐tunes these processes, modulating virulence and bacterial fitness during infection of woody olive plants. Together, these findings position the WHOP region as a crucial integrative hub that couples metabolic detoxification with adaptive traits essential for successful colonisation and persistence in woody hosts. By bridging biochemical pathways and complex phenotypes, the WHOP region presents promising targets for innovative strategies to control bacterial diseases in economically important woody crops. Future research should explore how WHOP‐mediated pathways respond to the diverse array of indole‐containing defence metabolites produced by woody plants, including phytoalexins, lignin‐derived compounds and tryptophan‐related molecules.

## Author Contributions

**Antonio Arroyo‐Mateo:** methodology, software, data curation, investigation, validation, formal analysis, visualisation, writing – original draft preparation, writing – review and editing. **Jesús Leal‐López:** methodology, investigation, validation, formal analysis, visualisation. **Luis Rodríguez‐Moreno:** validation, supervision, funding acquisition, project administration, resources, writing – review and editing. **Cayo Ramos:** conceptualisation, methodology, data curation, validation, formal analysis, supervision, funding acquisition, visualisation, project administration, resources, writing – original draft preparation, writing – review and editing.

## Conflicts of Interest

The authors declare no conflicts of interest.

## Supporting information


**Figure S1:** Mapping efficiency of RNA‐seq reads to the *P. savastanoi* pv. savastanoi NCPPB 3335 genome. Bar chart showing the number of quality‐filtered reads mapped to the genome of *P. savastanoi* pv. savastanoi NCPPB 3335 using Bowtie 2 (Langmead and Salzberg 2012). The dataset includes three biological replicates of the wild‐type (WT) strain (*n* = 3) and three replicates of the Δ*whpR* mutant (*n* = 3). Uniquely mapped paired‐end (PE) reads are shown in blue, and multi‐mapped PE reads in orange. Read counts are indicated in thousands (k).

**Figure S2:** Quality control and exploratory analysis of *P. savastanoi* pv. savastanoi RNA‐seq samples. (A) Boxplot showing the distribution of normalised gene expression levels in three biological replicates of the wild‐type (WT) strain *P. savastanoi* pv. savastanoi NCPPB 3335 (*n* = 3) and three of the Δ*whpR* mutant (*n* = 3). (B) Principal component analysis (PCA) based on normalised gene expression data. Wild‐type samples are enclosed by a blue ellipse, and Δ*whpR* samples by an orange ellipse. Principal component 1 (PC1) explains 40% of the variance, and principal component 2 (PC2) accounts for 16%.

**Table S1:** Strains used in this study.

**Table S2:** Plasmids used in this study.

**Table S3:** Primers used in this work and their applications.

**Table S4:** Bacterial genomes encoding WHOP‐like clusters identified in this study.

**Table S5:** RNA‐seq gene count matrix of the *P. savastanoi* pv. savastanoi NCPPB 3335 genome.

**Table S6:** KEGG‐predicted enzymatic functions and pathways associated with WHOP‐encoded and WhpR‐regulated genes.

**Table S7:** Differentially expressed genes in the *P. savastanoi* pv. savastanoi NCPPB 3335 Δ*whpR* mutant compared with the wild‐type strain.

## Data Availability

RNA‐seq data have been deposited in the Sequence Read Archive (SRA) at NCBI under accession number PRJNA1141921. All other data supporting the findings of this study are included in the Supporting Information or are available from the corresponding author upon reasonable request.
