# Supplementary material for: AraC‐Family Transcriptional Regulator WhpR Controls Virulence in Pseudomonas savastanoi pv. savastanoi Through Regulation of Indole Metabolism
Source: Microb Biotechnol. 2025 Oct 21;18(10):e70247. doi: 10.1111/1751-7915.70247 (PMC12538310; doi:10.1111/1751-7915.70247)
Supplement: Supplementary file 1 — Figure S1: Mapping efficiency of RNA‐seq reads to the P. savastanoi pv. savastanoi NCPPB 3335 genome. Bar chart showing the number of quality‐filtered reads mapped to the genome of P. savastanoi pv. savastanoi NCPPB 3335 using Bowtie 2 (Langmead and Salzberg 2012). The dataset includes three biological replicates of the wild‐type (WT) strain (n = 3) and three replicates of the ΔwhpR mutant (n = 3). Uniquely mapped paired‐end (PE) reads are shown in blue, and multi‐mapped PE reads in orange. Read counts are indicated in thousands (k). [file MBT2-18-e70247-s009.pdf]

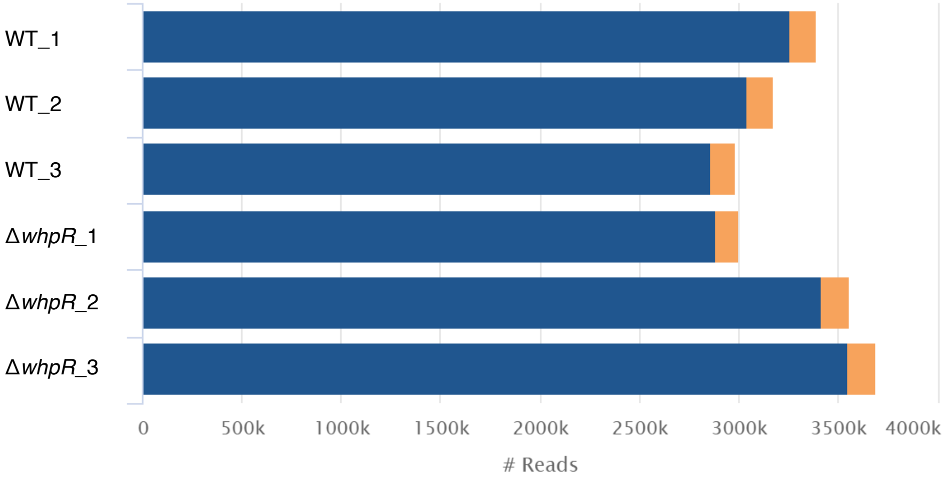

FIGURE S1. Mapping efficiency of RNA-seq reads to the *P. savastanoi* pv. savastanoi NCPPB 3335 genome. Bar chart showing the number of quality-filtered reads mapped to the genome of *P. savastanoi* pv. savastanoi NCPPB 3335 using Bowtie 2 (Langmead & Salzberg, 2012). The dataset includes three biological replicates of the wild-type (WT) strain (n = 3), and three replicates of the Δ*whpR* mutant (n = 3). Uniquely mapped paired-end (PE) reads are shown in blue, and multi-mapped PE reads in orange. Read counts are indicated in thousands (k).

References

Langmead, B. and Salzberg, S.L. (2012) Fast gapped-read alignment with Bowtie 2. *Nat Methods* **9**: 357–359.
