# Supplementary material for: AraC‐Family Transcriptional Regulator WhpR Controls Virulence in Pseudomonas savastanoi pv. savastanoi Through Regulation of Indole Metabolism
Source: Microb Biotechnol. 2025 Oct 21;18(10):e70247. doi: 10.1111/1751-7915.70247 (PMC12538310; doi:10.1111/1751-7915.70247)
Supplement: Supplementary file 2 — Figure S2: Quality control and exploratory analysis of P. savastanoi pv. savastanoi RNA‐seq samples. (A) Boxplot showing the distribution of normalised gene expression levels in three biological replicates of the wild‐type (WT) strain P. savastanoi pv. savastanoi NCPPB 3335 (n = 3) and three of the ΔwhpR mutant (n = 3). (B) Principal component analysis (PCA) based on normalised gene expression data. Wild‐type samples are enclosed by a blue ellipse, and ΔwhpR samples by an orange ellipse. Principal component 1 (PC1) explains 40% of the variance, and principal component 2 (PC2) accounts for 16%. [file MBT2-18-e70247-s007.pdf]

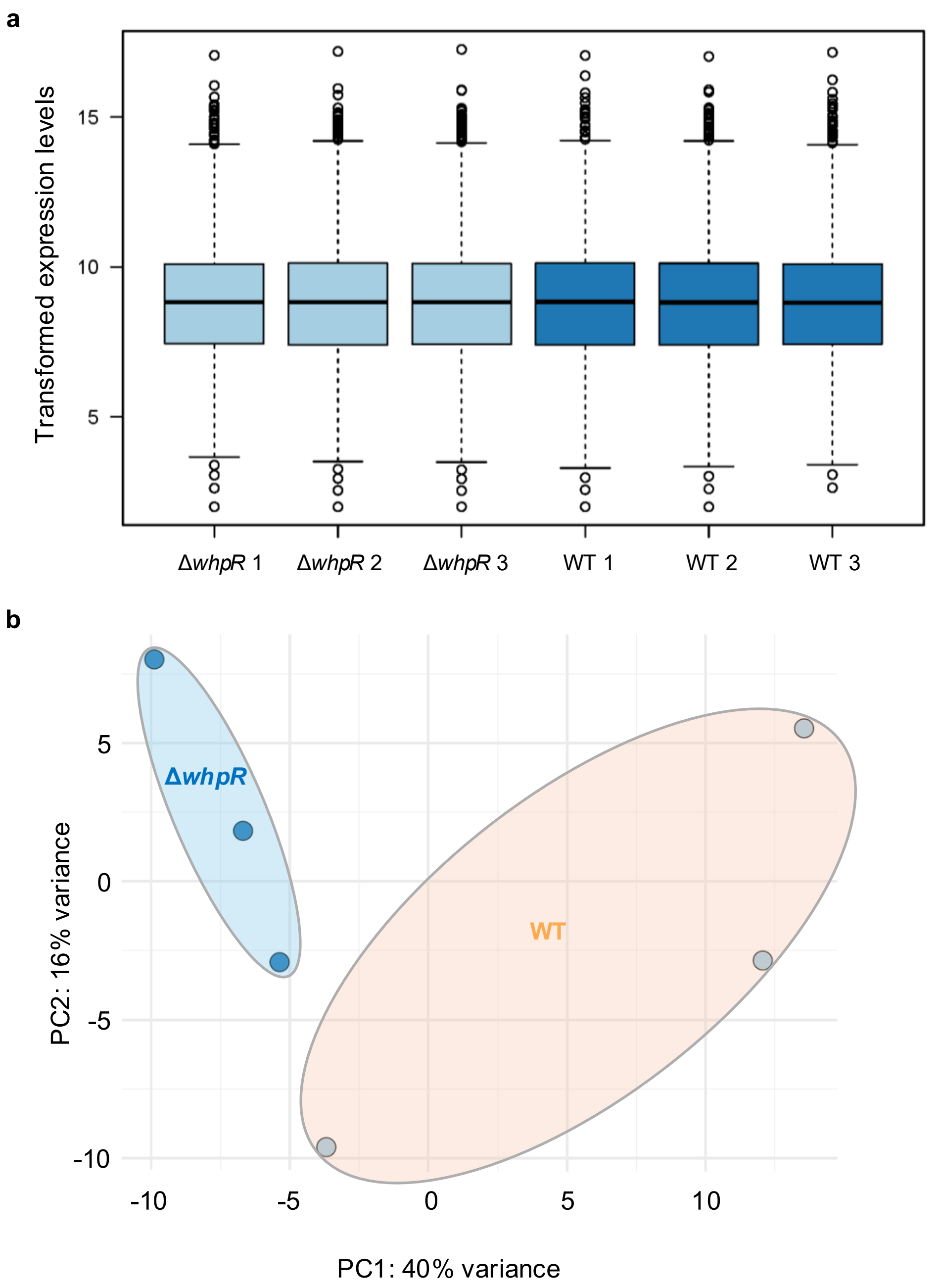

FIGURE S2. Quality control and exploratory analysis of *P. savastanoi* pv. savastanoi RNA-seq samples. (A) Boxplot showing the distribution of normalised gene expression levels in three biological replicates of the wild-type (WT) strain *P. savastanoi* pv. savastanoi NCPPB 3335 (n =3) and three of the Δ*whpR* mutant (n = 3). (B) Principal component analysis (PCA) based on normalized gene expression data. Wild-type samples are enclosed by a blue ellipse, and Δ*whpR* samples by an orange ellipse. Principal component 1 (PC1) explains 40% of the variance, and principal component 2 (PC2) accounts for 16%.
