# Supplementary material for: AraC‐Family Transcriptional Regulator WhpR Controls Virulence in Pseudomonas savastanoi pv. savastanoi Through Regulation of Indole Metabolism
Source: Microb Biotechnol. 2025 Oct 21;18(10):e70247. doi: 10.1111/1751-7915.70247 (PMC12538310; doi:10.1111/1751-7915.70247)
Supplement: Supplementary file 3 — Table S1: Strains used in this study. [file MBT2-18-e70247-s006.pdf]

TABLE S1. Strains used in this study

Strain	Main features[a]	Reference
***Pseudomonas savastanoi* pv. savastanoi**		
NCPPB 3335	Wild-type strain isolated from olive	(Pérez-Martínez et al., 2007)
Δ*whpR*	*whpR* (PSA3335_RS13035) deletion mutant (Km^R)	This work
Δ*dhoAB*	*dhoAB* operon deletion mutant (Km^R)	(Caballo-Ponce et al., 2017)
Δ*ipoABC*	*ipoABC* operon deletion mutant (Km^R)	(Caballo-Ponce et al., 2017)
Δ*ipo::ipo*	*ipoABC* operon deletion mutant complemented with pBBR:*ipoABC* (Gm^R)	(Caballo-Ponce et al., 2017)
Δ*whpR::whpR*	Δ*whpR* derived strain complemented with the Psv NCPPB 3335 *whpR* gene using pAMEX:*whpR* (Km^R)	This work
Escherichia coli		
Dh5α	F−, f80d*lac*ZΔM15, Δ(*lac*ZYA-*arg*F) U169, *deo*R, *rec*A1 *end*A1, *hsd*R17(rK− mK+), *pho*A, *sup*E44, λ−, *thi*-1, *gyr*A96, *rel*A1	(Hanahan, 1983)
GM2929	F- *ara-14 leuB6 thi-1 tonA31 lacY1 tsx-78 galK2 galT22 glnV44 hisG4 rpsL136, xyl-5 mtl-1 dam13::Tn9 dcm-6 mcrB1 hsdR2 mcrA recF143*. (Str^R Cm^R)	(Palmer and Marinus, 1994)

[a]Km^R, Gm^R, Str^R, Cm^R, kanamycin, gentamycin, streptomycin, and chloramphenicol resistance, respectively

References

Caballo-Ponce, E., Van Dillewijn, P., Michaela Wittich, R., and Ramos, C. (2017) WHOP, a genomic region associated with woody hosts in the *Pseudomonas syringae* complex contributes to the virulence and fitness of *Pseudomonas savastanoi* pv . savastanoi in olive plants. *Mol Plant-Microbe Interact* **30**: 113–126.

Hanahan, D. (1983) Studies on transformation of *Escherichia coli* with plasmids. *J Mol Biol* **166:** 557–580.

Palmer, B. R., and Marinus, M.G. (1994) The *dam* and *dcm* strains of *Escherichia coli* — a review. *Gene* 143: 1–12.

Pérez-Martínez, I., Rodriguez-Moreno, L., Matas, I.M., and Ramos, C. (2007) Strain selection and improvement of gene transfer for genetic manipulation of *Pseudomonas savastanoi* isolated from olive knots. *Res Microbiol* **158**: 60–69.
