# Supplementary material for: AraC‐Family Transcriptional Regulator WhpR Controls Virulence in Pseudomonas savastanoi pv. savastanoi Through Regulation of Indole Metabolism
Source: Microb Biotechnol. 2025 Oct 21;18(10):e70247. doi: 10.1111/1751-7915.70247 (PMC12538310; doi:10.1111/1751-7915.70247)
Supplement: Supplementary file 4 — Table S2: Plasmids used in this study. [file MBT2-18-e70247-s008.pdf]

TABLE S2. Plasmids used in this study

Name	Description[a]	Reference
pFLP2	Contains the *flp* recombinase gene for excision of sequences flanked by FRT (FLP Recognition Target) sites via site-specific recombination.	(Hoang et al., 1998)
pAMEX	Broad-host-range expression vector with the *nptII* promoter (Km^R).	(Macho et al., 2009)
pAMEX:*whpR*	pAMEX derivative, containing the Psv NCPPB 3335 *whpR* gene (PSA3335_RS13035) flanked by Hind III and Xba I sequences (Km^R).	This work
pBBR1MCS-5	Broad host-range cloning vector (Gm^R).	(Kovach et al., 1995)
pBBR:*ipoABC*	pBBR1MCS-5 derivative containing the Psv NCPPB 3335 *ipoABC* operon and its promoter region flanked by HindIII restriction sites (Gm^R).	This work
pGEM-T Easy	Cloning vector containing *ori* f1 and *lacZ* (Amp^R).	Promega Cor., Madison, EEUU
p*whpR*-Km	pGEM-T Easy derivative containing 1 kb on each side of the *whpR* gene from Psv NCPPB 3335, interrupted by a Km^R gene (Amp^R, Km^R).	This work
pGEM-T-KmFRT-*BamH*I	Contains the Km^R gene from pKD4 and BamHI sites (Amp^R, Km^R).	(Ortiz-Martín et al., 2010)

[a]Km^R, Gm^R and Amp^R, kanamycin, gentamicin and ampicillin resistance, respectively.

References

Hoang, T.T., Karkhoff-Schweizer, R.R., Kutchma, A.J., and Schweizer, H.P. (1998) A broad-host-range Flp-FRT recombination system for site-specific excision of chromosomally-located DNA sequences: application for isolation of unmarked *Pseudomonas aeruginosa* mutants. *Gene* **212:** 77–86.

Kovach, M. E., Elzer, P.H., Hill, D. S., Robertson, G.T., Farris, M.A., Roop 2nd, R. M., et al. (1995) Four new derivatives of the broad-host-range cloning vector pBBR1MCS, carrying different antibiotic-resistance cassettes. *Gene* **166:** 175-176.

Macho, A.P., Ruiz-Albert, J., Tornero, P., and Beuzón, C.R. (2009) Identification of new type III effectors and analysis of the plant response by competitive index. *Mol Plant Pathol* **10:** 69–80.

Ortiz-Martín, I., Thwaites, R., Mansfield, J. W., and Beuzón, C.R. (2010) Negative Regulation of the Hrp Type III Secretion System in *Pseudomonas syringae* pv. phaseolicola. *Mol Plant-Microbe Interact* **23:** 682–701.
