# Supplementary material for: AraC‐Family Transcriptional Regulator WhpR Controls Virulence in Pseudomonas savastanoi pv. savastanoi Through Regulation of Indole Metabolism
Source: Microb Biotechnol. 2025 Oct 21;18(10):e70247. doi: 10.1111/1751-7915.70247 (PMC12538310; doi:10.1111/1751-7915.70247)
Supplement: Supplementary file 5 — Table S3: Primers used in this work and their applications. [file MBT2-18-e70247-s001.pdf]

TABLE S3. Primers used in this work and their applications

Application	Name	Sequence (5' - 3')
Deletion of the *whpR* gene	WhpR_F1	ACTCATGGCTCGAGGCCACAGTGCGTTCATGC
	WhpR_R1	AAGCTTGACTCACTATAGGGTGGCAACTCTCACTCGTGC
	WhpR_F2	CCCTATAGTGAGTCAAGCTTGCGCTTTCGTCAATGAGG
	WhpR_R2	ACTCATGGGAATTCAAGCCTGATGGCGTAATGC
Complementation of *whpR*	Whrp_F3	ACTCATAAGCTTATAAAGACTCAAGCTGCC
	Whrp_R3	ACTCATTCTAGACTATCCTCATTGACGAAA
Real time quantitative PCR	WhpR_qRT_F	TGAAGCGACGCTGATCAATG
	WhpR_qRT_R	AACTCTTCGGTGCGTTTGAC
	TrpA_qRT_F	GTGCGTTACGTACACCTTCG
	TrpA_qRT_R	CACCGAGCACGTTACTGAAG
	TrpB_qRT_F	TTCAACGCCGATGATTTCTA
	TrpB_qRT_R	CAACGTCGATGACACCTTCT
	AntA_qRT_R	CTCACCCTTGACGCCCAG
	AntA_qRT_F	CGCAACCTGAACATCTACCC
	CatB_qRT_F	TGACGGCATTATCGGTATCG
	CatB_qRT_R	CGTTGAGGTTGGTGGCG
	AntR_qRT_F	GCTGGACGAGTTGCTGGAG
	AntR_qRT_R	GCGAGAGAATCAAGCCTTTG
	Dho_qRT_F	AAGGCGGCGATCAAACAATT
	Dho_qRT_R	ACTGACCTCGGCGATTACC
