# Supplementary material for: AraC‐Family Transcriptional Regulator WhpR Controls Virulence in Pseudomonas savastanoi pv. savastanoi Through Regulation of Indole Metabolism
Source: Microb Biotechnol. 2025 Oct 21;18(10):e70247. doi: 10.1111/1751-7915.70247 (PMC12538310; doi:10.1111/1751-7915.70247)
Supplement: Supplementary file 8 — Table S6: KEGG‐predicted enzymatic functions and pathways associated with WHOP‐encoded and WhpR‐regulated genes. [file MBT2-18-e70247-s004.pdf]

**TABLE S6.** KEGG-predicted enzymatic functions and pathways associated with WHOP-encoded and WhpR-regulated genes

| *Gene* Locus tag[a] | Protein ID[b] | KEGG[c] | | | |
|---|---|---|---|---|---|
| | | **EC number (% identity)[d]** | **KO number** | **KEGG annotation** | **KEGG pathways** |
| **WHOP-Encoded genes** | | | | | |
| *whpR* PSA3335_RS13035 | WP_031595095.1 | NF | K04033 | NF | NF |
| ***dhoA*** **PSA3335_RS13040** | WP_031595093.1 | EC:3.1.1.45 | K01061 | Carboxymethylene-butenolide hydrolase | Degradation of chlorocyclohexane, chlorobenzene, fluorobenzoate, and toluene |
| *dhoB* PSA3335_RS13045 | WP_002554211.1 | EC:1.1.1.175 (34%) | K22185 | Putative short-chain dehydrogenase/reductase | Pentose and glucuronate interconversions |
| *ipoA* PSA3335_RS13050 | WP_031595090.1 | EC:1.14.14.11 (28%) | K14481 | Putative monooxygenase | Styrene degradation |
| *ipoB* PSA3335_RS13055 | WP_002554209.1 | EC:1.5.1.36 (46%) | K23470 | Flavin-reductase domain protein | Tyrosine metabolism<br>Riboflavin metabolism |
| *ipoC* PSA3335_RS13060 | WP_005733721.1 | NF | NF | NF | NF |
| PSA3335_RS13065 | WP_031595087.1 | NF | K03776 | Aerotaxis receptor | Two-component system<br>Bacterial chemotaxis |
| *antR* PSA3335_RS13070 | WP_002554206.1 | NF | K04033 | Ethanolamine operon regulatory protein | NF |
| ***antA*** **PSA3335_RS13075** | WP_161795778.1 | EC:1.14.12.1 | K05599 | Anthranilate 1,2-dioxygenase large subunit | Anthranilate degradation to catechol |

| | | | | | |
|---|---|---|---|---|---|
| | | | | (deaminating, decarboxylating) | |
| ***antB*** **PSA3335_RS13080** | WP_002554204.1 | EC:1.14.12.1 | K05600 | Anthranilate 1,2-dioxygenase small subunit (deaminating, decarboxylating) | Anthranilate degradation to catechol |
| ***antC*** **PSA3335_RS13085** | WP_002554203.1 | EC:1.18.1.- | K11311 | Anthranilate 1,2-dioxygenase reductase component | Anthranilate degradation to catechol |
| ***catB*** **PSA3335_RS13090** | WP_002554202.1 | EC:5.5.1.1 | K01856 | Muconate cycloisomerase | Catechol ortho-cleavage to 3-oxoadipate |
| ***catC*** **PSA3335_RS13095** | WP_002554201.1 | EC:5.3.3.4 | K03464 | Muconolactone D-isomerase | Catechol ortho-cleavage to 3-oxoadipate |
| ***catA*** **PSA3335_RS13100** | WP_005733729.1 | EC:1.13.11.1 | K03381 | Catechol 1,2-dioxygenase | Catechol ortho-cleavage to 3-oxoadipate |
| **WhpR-regulated genes encoded outside the WHOP region** | | | | | |
| ***trpA*** **PSA3335_RS00220** | WP_005732766.1 | EC:4.2.1.20 | K01695 | Tryptophan synthase alpha chain | Glycine, serine, and threonine metabolism Phenylalanine, tyrosine, and tryptophan biosynthesis |
| ***trpB*** **PSA3335_RS00225** | WP_002551347.1 | EC:4.2.1.20 | K01696 | Tryptophan synthase beta chain | Glycine, serine, and threonine metabolism Phenylalanine, tyrosine, and tryptophan biosynthesis |
| ***rutD*** **PSA3335_RS09345** | WP_002552193.1 | EC:3.5.1.- | K09023 | Pyrimidine utilization protein D | Pyrimidine metabolism |

[a]Genes in boldface are deregulated in the Δ*whpR* mutant of *P. savastanoi pv. savastanoi* NCPPB 3335 compared with the wild-type strain (see Table S7).
[b]Reference sequence identifiers are from the National Center for Biotechnology Information (NCBI).
[c]Kyoto Encyclopedia of Genes and Genomes (KEGG); EC, Enzyme Commission; KO, KEGG Orthology.
[d]Amino acid sequence identity with EC proteins; NF, not found.
